# High-Resolution Imaging and Morphological Phenotyping of *C. elegans* through Stable Robotic Sample Rotation and Artificial Intelligence-Based 3-Dimensional Reconstruction

**DOI:** 10.34133/research.0513

**Published:** 2024-10-30

**Authors:** Peng Pan, Pengsong Zhang, Sharanja Premachandran, Ran Peng, Shaojia Wang, Qigao Fan, Yu Sun, John A. Calarco, Xinyu Liu

**Affiliations:** ^1^ Department of Mechanical and Industrial Engineering, University of Toronto, Toronto, Ontario M5S 3G8, Canada.; ^2^ Department of Cell & Systems Biology, University of Toronto, Toronto, Ontario M5S 3G5, Canada.; ^3^ College of Marine Engineering, Dalian Maritime University, Dalian 116026, China.; ^4^ School of Internet of Things Engineering, Jiangnan University, Wuxi 214122, China.; ^5^ Institute of Biomedical Engineering, University of Toronto, Toronto, Ontario M5S 3G9, Canada.

## Abstract

Accurate visualization and 3-dimensional (3D) morphological profiling of small model organisms can provide quantitative phenotypes benefiting genetic analysis and modeling of human diseases in tractable organisms. However, in the highly studied nematode *Caenorhabditis elegans,* accurate morphological phenotyping remains challenging because of notable decrease in image resolution of distant signal under high magnification and complexity in the 3D reconstruction of microscale samples with irregular shapes. Here, we develop a robust robotic system that enables the contactless, stable, and uniform rotation of *C. elegans* for multi-view fluorescent imaging and 3D morphological phenotyping via the precise reconstruction of 3D models. Contactless animal rotation accommodates a variety of body shapes and sizes found at different developmental stages and in mutant strains. Through controlled rotation, high-resolution fluorescent imaging of *C. elegans* structures is obtained by overcoming the limitations inherent in both widefield and confocal microscopy. Combining our robotic system with machine learning, we create, for the first time, precise 3D reconstructions of *C. elegans* at the embryonic and adult stages, enabling 3D morphological phenotyping of mutant strains in an accurate and comprehensive fashion. Intriguingly, our morphological phenotyping discovered a genetic interaction between 2 RNA binding proteins (UNC-75/CELF and MBL-1/MBNL), which are highly conserved between *C. elegans* and humans and implicated in neurological and muscular disorders. Our system can thus generate quantitative morphological readouts facilitating the investigation of genetic variations and disease mechanisms. More broadly, our method will also be amenable for 3D phenotypic analysis of other biological samples, like zebrafish and *Drosophila* larvae.

## Introduction

Due to its short life cycle, ease of maintenance, transparency, well-characterized tissues and nervous system [1,2], and many genes similar to those of humans [3,4], the nematode *Caenorhabditis elegans* represents a widely employed model organism in biological studies [5–9]. In particular, the ease of live microscopy in *C. elegans* has deepened our understanding of cellular mechanisms governing neuronal development and function through visualization of phenotypes impacting neuronal morphology [10–13], neuronal gene expression [6], and 3-dimensional (3D) morphologies [14,15].

As worms at different developmental stages, including the embryonic stage, 4 larval stages (L1 to L4), and adulthood, possess different features and morphological properties, accurate visualization and 3D morphological phenotyping of worms can be utilized to measure subtle but statistically significant changes in organism size and shape, which could facilitate the discovery of new genetic mechanisms. More specifically, considering the important potential that *C. elegans* hold for genetic studies, clear visualization and comprehensive morphological phenotyping at different developmental stages can provide important complementary insights into animal development, especially when coupled with analysis of mutants [14,16].

Although there have been numerous innovations in microscopy techniques including confocal microscopy and volume electron microscopy [17], a reliance on any imaging method alone is often insufficient for the precise and efficient 3D morphological phenotyping of *C. elegans*. Challenges arising from imaging can include worm distortion during the preparation process, imaging artifacts, irreversible damage caused to animals, the requirement of expensive equipment, and time-consuming and complex operations [17–20]. Thus, the introduction of a uniform and controllable rotation method, in conjunction with regular widefield microscopy, would serve to gather 3D contour data comprehensively and precisely at desired orientations, providing a simple yet effective alternative to more sophisticated microscopy techniques for precise and thorough 3D morphological phenotyping. In addition, through the controlled rotation of worms to distinct orientations, high-resolution fluorescent imaging of *C. elegans* anatomical and cellular structures can be obtained by overcoming some of the limitations of imaging of *C. elegans* by both widefield (obscured features) and confocal microscopy (i.e., notable decrease in intensity and resolution of signal away from the coverslip under high magnification) [21].

Despite the utility of many developed approaches for the rotational manipulation at the single cell level (mainly including optical tweezer [22], magnetic means [23], electrical means [24,25], and acoustic means [26]), methods to achieve controlled *C. elegans* rotation are limited. Recently, a glass capillary has been used to capture and rotate the head of a worm loaded inside microchannels [27]. However, capturing worms by using this approach is difficult. In another method, worms are passively oriented by a curved channel geometry during loading [28], but the loaded worms can only be rotated to a certain angle. In these 2 rotation methods, worms are severely squeezed, which hinders accurate morphological phenotyping. Bulk acoustic waves [29–31] and surface acoustic wave (SAW) [32] have been employed to generate a vortex for the rotation of *C. elegans*. For the acoustic wave-based methods, several limitations hinder the precise and efficient 3D morphological phenotyping of *C. elegans*. These limitations include localized rotation, inconsistent bubble size and geometry resulting in nonuniform rotation, the obstruction of worms induced by the contact between rotated worms and external structures, the necessity for specific substrates possessing large thickness, low transmittance, and a large refractive index preventing high-quality imaging. Moreover, all the methods mentioned above are inappropriate for the reliable and controllable rotation of worms at various developmental stages. In this regard, there is an unmet need to develop a contactless, precise, and uniform worm rotation method that is independent of the substrate and can be used for measurements across animal development.

Herein, we demonstrate a robotic system integrating a microfluidic device, which enables reliable, controllable, and uniform rotation of worms at different developmental stages for clear visualization of different features and precise 3D morphological phenotyping in a contactless manner. Leveraging the laminar flow from the microchannels of a microfluidic device and the frictional force between the worm body and the substrate, contact-free rotational manipulation of animals is obtained with a consistent gap between worms and the open ends of microfluidic device. We demonstrate that worms at different developmental stages ranging from embryos to adulthood can be stably rotated at a low speed, enabling the capture of a larger set of 2D images within a single rotation period. It is important to note that multiple samples, such as 2 adults or 2 embryos, can be simultaneously rotated. As proof-of-concept experiments, we performed high-resolution imaging of transgenic worms with fluorescence-marked features at distinct orientations via controlled rotation.

Additionally, because of the uniform and stable worm rotation, we create, for the first time, dense 3D models of *C. elegans* at the embryonic and adult stages, based on the large collection of 2D images within a single rotation period. The resultant 3D models are quantified as digital readouts that enable 3D morphology-based phenotypic analysis in an accurate and comprehensive manner. We validate our technology by phenotyping the 3D morphologies of embryos and young adults, which are the earliest and latest stages of *C. elegans*, respectively, from wild-type and several mutant strains. Intriguingly, we discover a genetic interaction between 2 conserved RNA binding proteins (encoded by *unc-75* and *mbl-1*) indicated by changes in the surface area and the length of mutant embryos as well as the changes in the ratio of length to maximum width of mutant adults compared with wild-type animals. Taken together, the consistent and accurate information gathered from the reconstructed 3D models using our technology can facilitate the understanding of complex genetic traits, diseases, and biological pathways. We believe that the proposed rotation method also holds a huge potential in the 3D morphological phenotyping and high-resolution imaging of other biological samples, such as zebrafish and *Drosophila* larvae, for various biological and biomedical applications, including accelerating genetic interaction analysis, quantitative trait profiling, and drug screening.

## Results

### Design of the robotic system for worm rotation

**Fig. 1.**
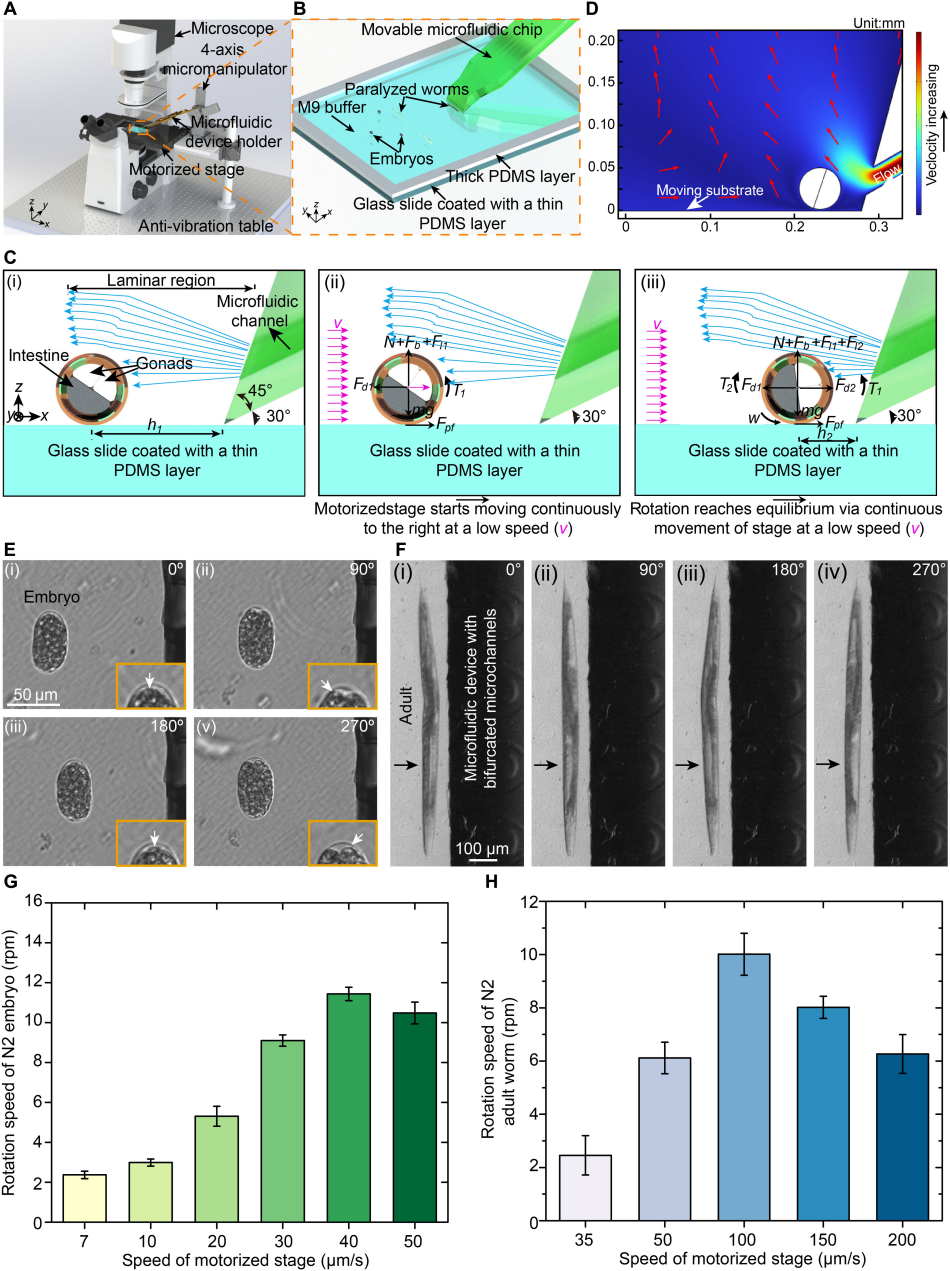
System setup for stable rotation of *C. elegans* and its embryos and high-resolution imaging of the rotated sample at different orientations. (A) Robotic system setup. (B) A microfluidic device and a sample reservoir (filled with embryos, larvae, or adult worms) for sample rotation. (C) Working principle of stable sample rotation. (D) General simulated streamline around the rotated sample when the substrate moves rightward and the fluid flows out of the open-ended channels. (E) Stable rotation of a single embryo. (F) Stable rotation of an adult worm. (G) Rotation speed of different embryos at the gastrulation stage as a function of the stage speed (*N* = 8). (H) Rotation speed of different adult worms as a function of the stage speed (*N* = 5).

To achieve controllable rotation of *C. elegans,* a robotic system was established, which consists of a motorized micromanipulator (MX7600, Siskiyou), a regular inverted microscope (IX83, Olympus), a microfluidic device, a custom-made reservoir, and a miniaturized pressure regulator (Fig. 1A and Fig. S1). The micromanipulator has 3 orthogonal and one diagonal motion axes with a step resolution of 40 nm and a motion range of 20 mm along each axis. The microfluidic device comprises a row of open-ended microfluidic channels, all connected to a computer-controlled pressure regulator, for robotic worm rotation (Fig. 1B and Fig. S2). To ensure equal fluidic resistances between the single device inlet and their open ends and thereby maintain uniform pressure at the open ends, the open-ended channels were organized in a bifurcation layout. Note that the microfluidic device was mounted with a tilting angle of 30° on the right micromanipulator. To avoid any image shading of the worm body by the device edge in the entire process of worm rotation, a smooth slope of 45° was then cut at the side wall of the microfluidic device where the open ends of the microchannels are arranged (Fig. S2). The detailed fabrication process of the microfluidic device can be found in Fig. S2. The fabrication details of the microfluidic device can be found in the Supplementary Materials. The custom-made reservoir, used for loading of worms at different developmental stages, was prepared by bonding a patterned polydimethylsiloxane (PDMS) layer to a 3″ × 2″ glass slide with a 14-μm-thick PDMS layer coated on its top side (Fig. 1B). The miniaturized pressure regulator (Fig. S1) consisting of 2 miniature solenoid valves (10-32 UNF Female, 12V DC, McMaster-Carr) and 2 vacuum pumps (SFE 12V Air Pump, Robotshop) was used to provide negative pressure inside the microchannels of the microfluidic device, allowing for the infusion of buffer into the microfluidic channels and the plastic tube connecting the microfluidic device to the pressure regulator.

To achieve reliable rotation of worms at different developmental stages, we proposed a straightforward yet effective method utilizing the developed robotic system. First, the plastic tube, running from the inlet of the microfluidic device to its highest point (Fig. S1C), and the microfluidic channels were filled with M9 medium by activating the miniaturized pressure regulator. Once the negative pressure was no longer applied by turning off the miniaturized pressure regulator, the M9 medium inside the microfluidic channels and the tube would slowly flow out of the microchannel open ends under the action of gravity (Fig. S3). When a worm was brought to the open ends of the microfluidic device by moving the motorized stage of the microscope at a small and constant speed, it would be continually subject to several forces, including the frictional force (*F_pf_*) between the worm body and the substrate, and hydrodynamic forces resulting from the fluid flowing out of the open-ended channels [Fig. 1C(ii)]. In combination with the 45° slope and the relatively greater height of the microfluidic channel center at the open ends, the hydrodynamic forces from the fluid flowing out of the microchannel open ends would mainly act on the top surface of the worm body (Fig. 1D and Figs. S4 and S5), resulting in the anti-clockwise rotation (Fig. 1C). Due to the low, constant speed of the motorized stage and the appropriate height of the plastic tube, the lift forces exerted on the worm body would also not be excessive, ensuring that rotated worms remain in focus. Thus, when coupled with microscopy, our proposed robotic system is amenable for the acquisition of high-quality images of animals at various orientations. The experimentally determined maximum height of the plastic tube was 5 cm (Fig. S1C). The detailed working principle of robotic rotation can be found in the Supplementary Materials.

### Rotation of embryos, larvae, and adult worms

The life cycle of *C. elegans* includes embryogenesis, 4 larval stages, and then adulthood [33]. At each of these stages, distinct features of *C. elegans,* such as varying morphology and size [34], differentiation and maturation of the nervous system [35], and dynamic changes in body composition [36], can be observed. Thus, the rotation and 3D morphological imaging of *C. elegans* at different developmental stages will provide detailed information of these features and thus facilitate the comprehensive understanding of complex genetic traits and biological pathways. Usually, the rotation angle of worms can be monitored by visually recognizing their body patterns (Fig. S6).

Rotational manipulation of the embryo was first demonstrated by utilizing our robotic system (Fig. 1E, Fig. S7, and Movies S1 and S2). When the motorized stage moved rightward at a constant speed (Fig. 1C), the embryo was first brought close to the open ends of the microfluidic channels by the frictional force between the embryo and the substrate, and self-aligned with the edge of the microfluidic device under the action of the frictional force and the hydrodynamic forces [Figs. S7 and S8 and Fig. 1E(i)]. Figure 1E(ii) shows the N2 embryo at gastrulation stage after being rotated by 90°, indicated by the movement of the transparent landmark from the middle position to the leftmost position inside the embryo (labeled by the white arrow in the enlarged view). After that, the embryo was further rotated to 180° and 270° [Fig. 1E(iii) and E(vi)] where the transparent landmark inside the embryo reached the middle position and the rightmost position, respectively. Consistent with our theoretical analysis mentioned above, smooth rotation was achieved and a constant gap between the embryo and the open-ended edge of the microfluidic device was maintained during the rotation process. In addition, under a constant stage speed of 10 μm/s, we found that rotating the same embryo by each 360° took 20.43 s ± 0.44 s (*N = 5*). The small standard deviation of the rotation time demonstrates that the rotation of the same embryo is uniform. In a separate trial, we also observed the simultaneous rotation of 2 embryos, again while maintaining a consistent gap between the animals and the microfluidic device (Fig. S7B). By visually monitoring the transparent landmark inside the embryos (labeled by the white arrow), rotation angles of 2 embryos can be recognized (Fig. S7B). These results demonstrate that the combination of frictional and hydrodynamic forces exerted on the embryo enables the stable rotation of one or even multiple embryos in a manner suitable for imaging at different orientation angles.

In addition to the embryos, we also used our system to rotate larvae and adult worms, which exhibit distinct features from embryos. To monitor the rotation angle of the worm body, we used easily recognized landmarks such as the transparent gonad and opaque intestinal cells in the brightfield mode. As was the case with embryo rotation, we successfully achieved a smooth, continuous rotation of adult worms indicated by the periodic change in the size of the bright area while maintaining a consistent gap between the worm and the microfluidic device (Fig. 1F and Movies S3 and S4). The combination of the consistent gap and consistently focused adult worm allows for the high-quality imaging of the worm body during the rotation process. Similarly, under a constant stage speed of 50 μm/s, we found that rotating the same adult worm by 360° took 11.06 s ± 0.24 s (*N = 5*) also indicating the uniform rotation of the same adult worm. Moreover, the smooth and continuous rotation of larvae, ranging from L2 to L4, was also achieved with a consistent gap between the worm and the open-ended channels of the microfluidic device, further demonstrating that the rotation is independent of body size (Figs. S7 and S8D to F and Movies S5 to S7). It is worth noting that despite the relatively curved shape of larval and adult animals, rotation was still reliable and independent of change in the worm body shape. Once a worm was rotated to the desired orientation, the stoppage of worm rotation could be quickly obtained by rapidly moving the worm to the left, away from the open-ended channels of the microfluidic device (Fig. S9 and Movie S8). Also, the rotation success rates of *C. elegans* at different developmental stages were measured, which are all 100% for embryos (*N* = 12), L2 (*N* = 9), L3 (*N* = 8), L4 (*N* = 9), and adults (*N* = 10).

Considering that rotation is dependent on the use of a motorized stage, we next experimentally tested the effect of the stage speed on rotation performance of embryos and adult worms, which have oval and rod-like shapes, respectively. The *C. elegans* embryo is small in size, and thus, it has a relatively low weight. To prevent the embryo from lifting during the rotation process, embryos at gastrulation stage were rotated with relatively small speeds of the motorized stage ranging from 7 to 50 μm/s (Fig. 1G). As the speed increased from 7 to 40 μm/s, the average rotation speed of different embryos increased with small standard deviations at different levels (Fig. 1G), showing that the embryo rotation was consistent and stable. However, upon increasing the speed to 50 μm/s, the embryo rotation speed decreased. Compared to the embryo, an adult worm is larger, and thus, it has a relatively greater weight. Therefore, different adult worms were rotated at speeds of the stage ranging from 35 to 200 μm/s (Fig. 1H). Again, the adult worm rotation speed increased at low motorized stage speeds ranging from 35 to 100 μm/s (Fig. 1H). However, when the stage speed increased to 150 and 200 μm/s, the adult worm rotation speed decreased. It should be noted at relatively high speeds of the motorized stage (50 to 200 μm/s), the rotation speed of adult worms exhibited small standard deviations, implying stable and consistent rotation of different adult worms. The relatively low rotation speeds achieved by our system (i.e., ~2 to 11 rpm) allow the capture of a larger set of 2D images within a single rotation period.

In summary, there are several advantages of the proposed worm rotation method. First, by moving *C. elegans* rightward at a relatively small constant speed, the frictional force and the hydrodynamic forces that are experienced by *C. elegans* would not be excessive, allowing for the stable and uniform rotation of worms in a slow fashion while ensuring that rotated worms remain in focus. The slow rotation offers superior control over worm manipulation and provides the advantage of capturing a large sequence of high-quality 2D images within a full rotation, which facilitates the high-resolution imaging and thus allows the precise 3D reconstruction of worms. In addition, a rotational torque is induced by the combined effect of the frictional force and the hydrodynamic forces, enabling contactless, precise, and stable rotation of *C. elegans*. This rotation mechanism is independent of body shape and body size. With its contactless nature and independence of the body shape/size, the proposed rotation method enables stable rotation of worms across different developmental stages and various mutant strains with varying body shapes/sizes. Moreover, unlike other rotation methods that rely on specific substrates, our worm rotation method is independent of the substrate, making it easily applicable and highly advantageous in many applications, especially those necessitating a thin coverslip as the sample substrate for high-resolution imaging under high magnification. In addition, the movable microfluidic device and the capability of simultaneous manipulation of multiple samples enable rotation in a successive fashion.

### Fluorescent imaging of *C. elegans* neurons and muscles

**Fig. 2.**
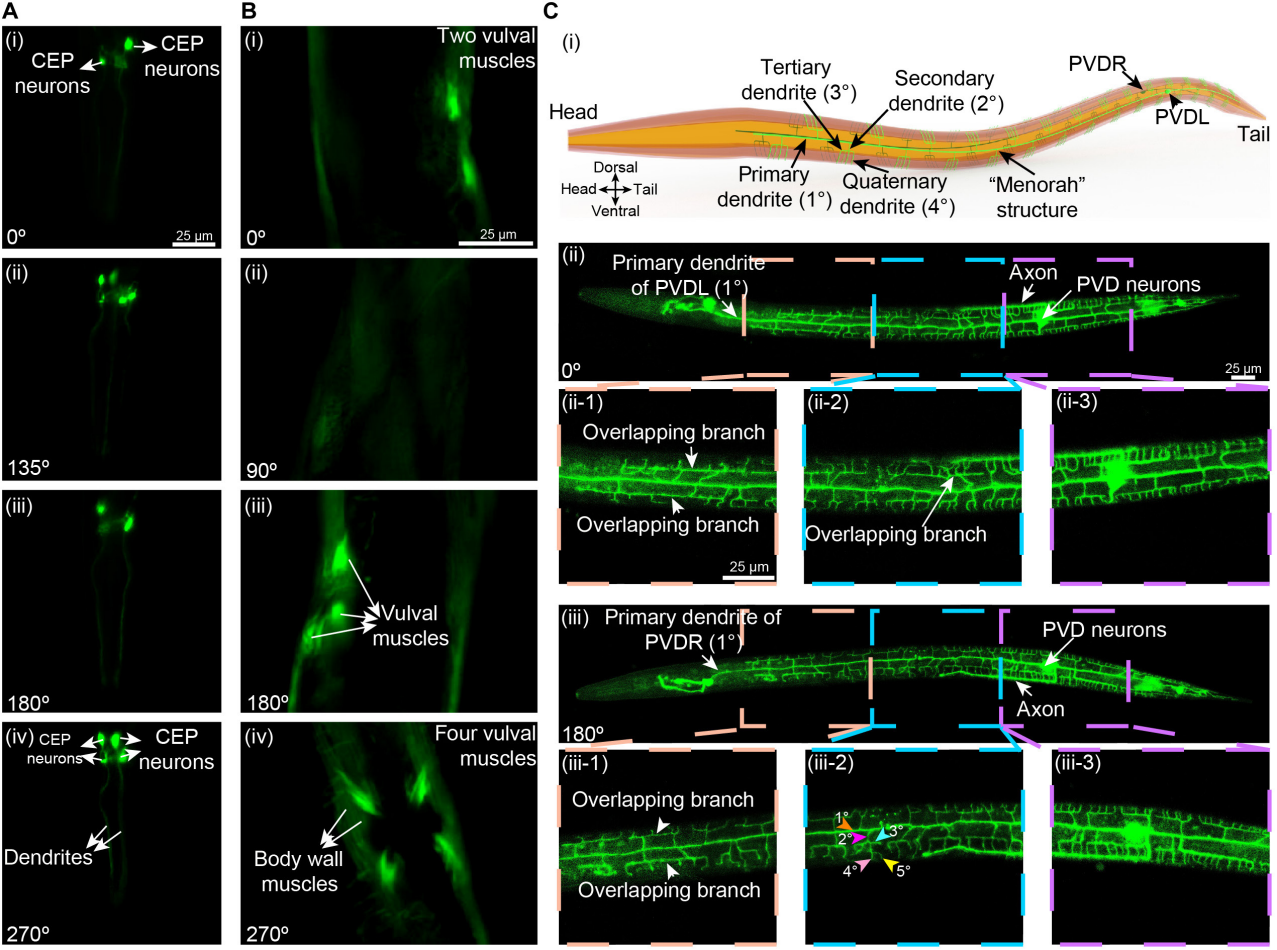
Fluorescent imaging of *C. elegans* neurons, muscles, and complex dendrites patterns at different worm body orientations realized by the developed robotic system. (A) Fluorescent imaging of the CEP neurons of *C. elegans* (TG2435) at different orientations. (B) Fluorescent imaging of vulva muscles of *C. elegans* (RW1596) at different orientations. (C) Fluorescent imaging of PVD dendrites of worm (NC1687) at the L4 stage at different orientations.

To demonstrate the ability of the developed robotic system to perform 3D imaging of *C. elegans*, we first used it to examine the specific neurons of a *C. elegans* strain (TG2435) expressing green fluorescent protein (GFP) in dopaminergic neurons driven by the *dat-*1 promoter. This transgene produces bright green fluorescence specifically in the 8 dopaminergic neurons including 4 cephalic (CEP) neurons, 2 anterior deirid (ADE) neurons, and 2 posterior deirid (PDE) neurons. The CEP neurons are distributed in the worm head. Conventionally, clear visualization of individual CEP neurons by widefield microscopy is difficult as these CEP neurons may overlap with one another, depending on the orientation of the worm head. In our experiments with controlled worm rotation, the fluorescent images of the CEP neurons were obtained at distinct orientations (Fig. 2A). At some rotation angles, due to the overlap in the horizontal plane of the cell bodies of some of the CEP neurons, only 2 of the 4 neurons were observed [Fig. 2A(i) and A(iii)]. However, when rotated through different angles, all 4 CEP neurons can be observed [Fig. 2A(ii) and A(iv)]. Especially, after rotating the worm by 270°, the CEP neurons were totally separated, and all CEP neurons can be clearly observed [Fig. 2A(iv)]. Also, fluorescent imaging of all head neurons in the anterior part of *C. elegans* (JAC769) from different orientations was shown in Fig. S10.

We also examined the body wall and vulval muscle cells of adult worms at distinct orientations using a transgenic strain (RW1596) expressing GFP driven by the *myo-3* promoter (Fig. 2B). Again, due to the overlap in the field of view of the widefield microscopy, only 2 vulva muscles were visible at certain rotation angles, and none was visible when viewed from the dorsal perspective [Fig. 2B(i) and B(ii)]. Once the worm was rotated to the orientation of 180°, the number of observed vulva muscles increased to 3 [Fig. 2B(iii)]. However, upon rotation to the orientation of 270°, which enables the visualization of the ventral side of the animal, all 4 vulval muscles were clearly visible [Fig. 2B(iv)]. Taken together, our robotic system offers a simple solution for overcoming the limitations of imaging of *C. elegans* by widefield fluorescent microscopy through controlled rotation to desired imaging angles.

### Fluorescent imaging of PVD dendrites

With a fully mapped connectome and its highly stereotyped structures, the *C. elegans* nervous system is used as an outstanding model for in vivo functional studies of neuronal development [37]. Neurons derived from the posterior V lineage (PVD) are a pair of bilateral multidendritic neurons that respond to harsh touch and extreme temperatures [38,39]. The PVD neurons undergo a progressive development of elaborate dendritic arborization patterns from the L2 to L4 stage [40], generating a series of orthogonal primary (1°), secondary (2°), tertiary (3°), and quaternary (4°) dendritic branches [37], and resulting in the formation of repeated “menorah” structures [as schematically shown in Fig. 2C(i)]. Due to their complex patterns and substantial sizes, the PVD neurons are ideal for the discovery of genetic mechanisms that regulate neurogenesis and neuronal physiology. Typically, the confocal microscopy is employed for the clear imaging of dendrites of worms, where worms are mounted on an agar plate with a lateral position [41–43]. However, the quality of fluorescent signals obtained in each *z*-slice from the confocal imaging experiences a notable decrease in intensity and resolution when imaging further away from the coverslip [21]. This is particularly apparent when relatively thick tissue samples such as *C. elegans* at late larval and adulthood stages are imaged under high magnifications. As a result, using confocal microscopy, clear imaging is achievable only for the dendrites of a PVD neuron closest to the objective (Fig. S11).

We reasoned that the employment of our robotic system can reduce the barrier to clear and comprehensive imaging of PVD neurons and the bilateral PVD left and right (PVDL and PVDR) dendrites distributed over the majority of worm body by using the confocal microscope. Indeed, utilizing a strain (NC1687) expressing GFP in the PVD neurons, we were able to clearly and comprehensively image both the left and right PVDs, both axons, and their full dendritic complement including quaternary and even quinary branches of menorahs, by using a confocal microscopy (A1R, Nikon) (Fig. 2C). As shown in Fig. 2C(ii), a worm at the L4 stage was first rotated to the 0° orientation in which the primary dendrite of PVDL neuron was located at the midline of the worm and its axon was located at the top side of the fluorescent image. Dendrites emanating from the neuron of PVDL over the majority of the worm body were clearly imaged. After the worm was rotated by 180°, we could clearly observe the primary dendrite of the PVDR neuron, which was located at the midline of the worm [Fig. 2C(iii)]. Also, its axon was located on the bottom side of fluorescent image [Fig. 2C(iii)]. Three enlarged views of each PVD dendrite were displayed in Fig. 2C(ii) and Fig. 2C(iii), respectively. From these fluorescent images, we can see that the repeated “menorah” structures of PVDL and PVDR neurons were different. Neuronal self-avoidance is an important property of various types of neurons including the PVD neuron of *C. elegans,* which displays robust self-avoidance between sister dendrites [44]*.* However, occasionally in wild-type animals, some sister dendrites do occasionally overlap. Accurately counting such phenotypes would benefit from observing animals at multiple perspectives. Accordingly, we were able to observe a lack of self-avoidance at several locations, both while viewing the right and left PVD of the same animal [Fig. 2C(ii) and C(iii)]. Usually, the terminal dendrites of PVD neuron are quaternary (4°) [Fig. 2C(i)]. However, a quinary dendrite (5°) was observed in Fig. 2C(iii-2). Finally, the terminal (quaternary and quinary) dendrites of PVDR neuron were counted to be 124 [Fig. 2C(ii)] and the number of terminal (quaternary and quinary) dendrites of PVDL was 152 [Fig. 2C(iii)]. Taken together, our robotic system allows for high-resolution imaging of the bilateral PVD dendrites of L4-stage animals, which provides a straightforward and effective solution for overcoming the imaging limitations of relatively thick samples such as *C. elegans* encountered with confocal microscopy. More broadly, the clear and comprehensive imaging enabled by our robotic system will facilitate more detailed mapping of neuronal morphology of *C. elegans*, with the ability to fully capture bilateral pairs of neurons from the same animal.

### Artificial intelligence-based 3D reconstruction of *C. elegans*

**Fig. 3.**
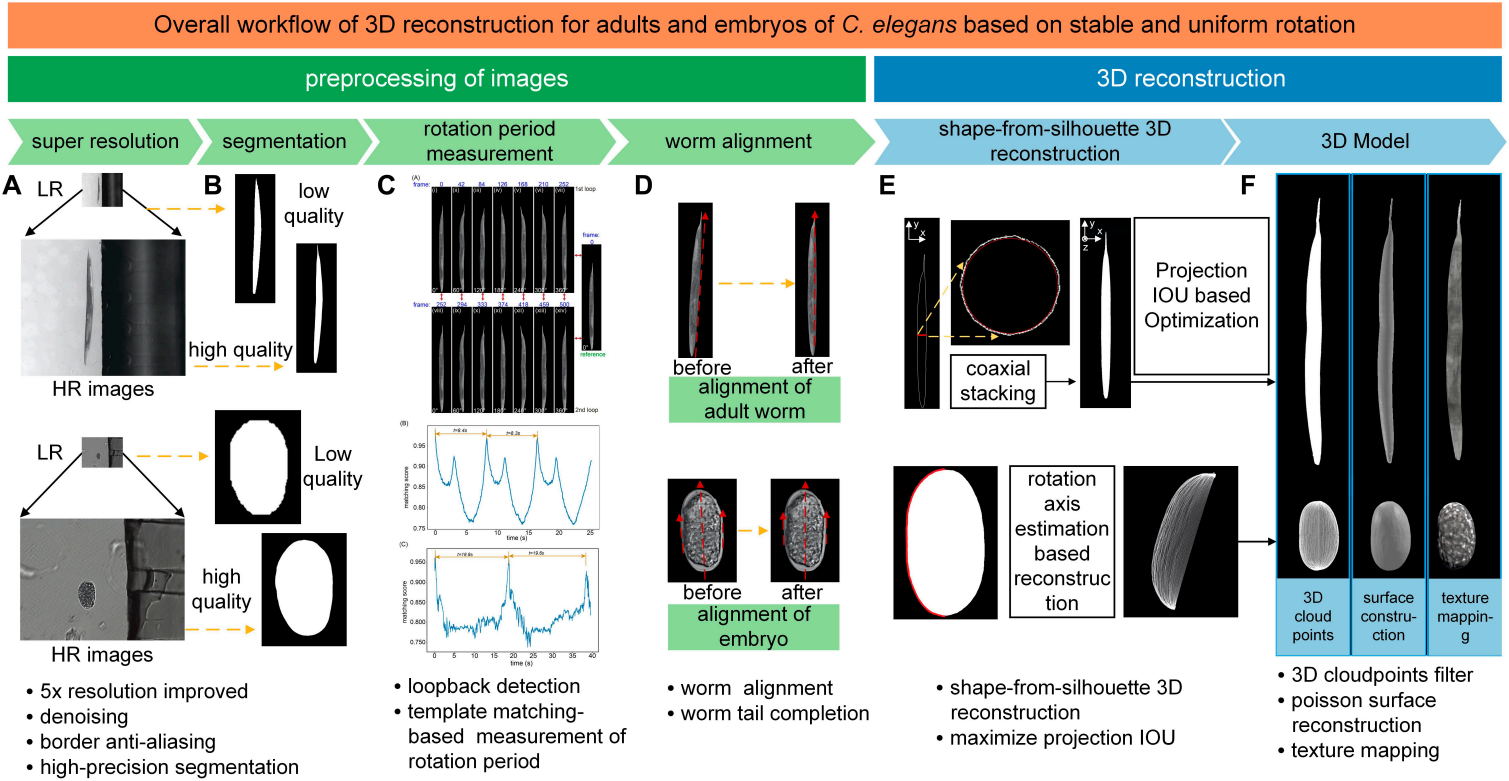
Overall workflow of the 3D reconstruction of *C. elegans*. (A) Enhancement of image quality by novel machine learning model-based super-resolution and denoising while the details of *C. elegans* in microscopy images are preserved. (B) Machine learning-based accurate segmentation of *C. elegans.* (C) Template matching algorithm-based accurate measurement of worm rotation speed. (D) Alignment of *C. elegans* body for precise 3D reconstruction thereafter. (E) 3D reconstruction of worms based on the customized shape-from-silhouette 3D reconstruction algorithms. (F) Generated 3D models of *C. elegans.*

Accurate and automated measurements of the 3D morphological properties of biological samples, such as cells and animals, can provide interesting quantitative traits to assess in the context of the genetics of morphology, shape, and size during development and disease [14,45,46]. However, the current method for 3D morphological phenotyping of *C. elegans* is limited and inaccurate [14]*.* Previous 3D morphological phenotyping of *C. elegans* relies solely on the inaccurate estimation of volume based on the contours extracted from a 2D image (Fig. S12)*,* which adversely affects the accuracy and consistency of genetic studies [14]. Because of the stable, uniform, and slow worm rotation achieved by our robotic system, we can create precise 3D reconstructions of *C. elegans* at different developmental stages based on a substantial number of 2D images captured within a single rotation period (Fig. 3 and Movies S9 and S10). The resultant 3D models of worms could be quantified as digital readouts enabling 3D morphology-based phenotypic analysis thereafter (e.g., volume, surface area, length, maximum width, and ratio of length to maximum width) in an accurate and comprehensive fashion.

Specifically, customized algorithms were developed to reconstruct the 2D images into 3D models of adult worms or embryos following a processing sequence of image super-resolution (Fig. 3A and Figs. S13 and S14), accurate worm detection and segmentation (Fig. 3B and Figs. S15 and S16), precise measurement of rotation period (Fig. 3C and Fig. S17), in-plane alignment of detected worm body/embryo and image feature completion (Fig. 3D, Figs. S18 and S19, and Table S1), and 3D reconstruction (Fig. 3E and F and Figs. S20 to S23).

Given that an adult worm has a maximum diameter of ~50 μm and a body length of ~1 mm, 2D bright-field images of rotated animals were captured under an 4× objective using conventional widefield microscopy. Due to the presence of background noise, the transparent body, and the low spatial resolution under the 4× objective [Fig. S21A(i)], the accurate segmentation of adults in each image is difficult. To begin, machine learning-based algorithms were customized to enhance the quality of captured 2D images [Fig. S21A(ii)] and realize accurate detection as well as segmentation of the worm in each high-resolution image [Fig. S21A(iii)]. During the worm rotation process, there existed small in-plane drift of the worm body. Thus, a customized algorithm was developed to align the detected worm body in each 2D image [Fig. S21A(iv)], and then segmentation of the aligned worm body in each 2D image was achieved [Fig. S21A(v)] for the precise 3D reconstruction thereafter (Fig. S21A and B). The accurate measurement of worm rotation speed was obtained by utilizing a template matching algorithm [47] (Fig. S17), facilitating the precise 3D reconstruction of worms at different developmental stages. Finally, the contours of aligned worm bodies in each 2D image captured within a single rotation period were processed for 3D reconstruction of the adult worm based on a customized shape-from-silhouette 3D reconstruction algorithm (Fig. S21B). Owing to its small size and transparency, the tail of an adult worm in bright-field images exhibits extremely low contrast when compared to the background, which makes the detection of the entire tail in each 2D image difficult (Figs. S18A and S21C). With the customized 3D reconstruction algorithms, the missing features of the tail at certain orientations can be effectively reconstructed (Figs. S18A and S21C).

**Fig. 4.**
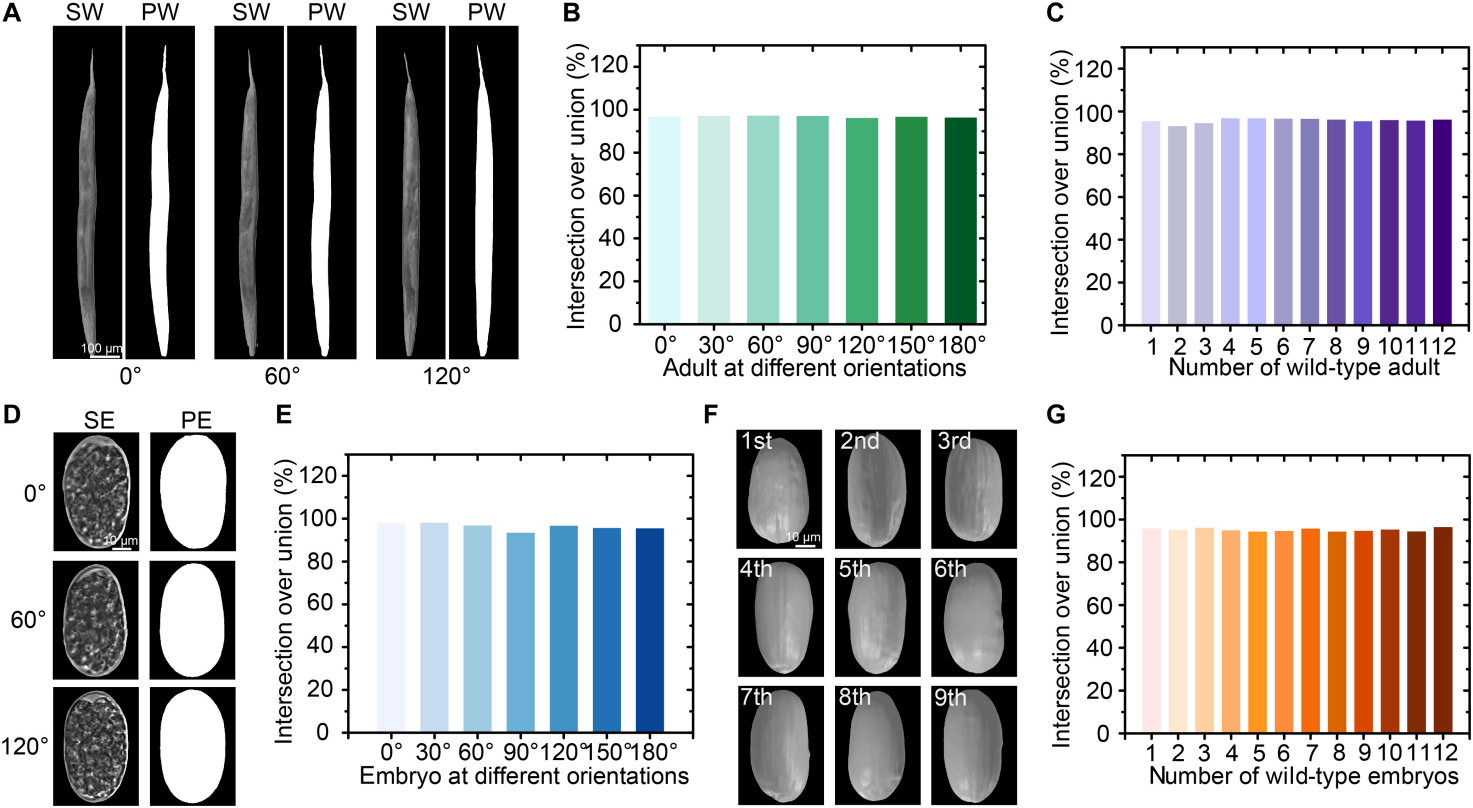
3D reconstruction of *C. elegans* from a large collection of 2D images*.* (A) Comparisons between the projected contours of 3D worm model and the segmented worms in the captured microscope image at the orientations of 0°, 60°, and 120° (SW, segmented worm; PW, projected worm contour). (B) IoU validation for the reconstructed 3D model of adult worm at different orientations. (C) IoU validation for the reconstructed 3D model of different adult worms. (D) Comparisons between the projected contours of 3D embryo model and the segmented embryo in the captured microscope image at the orientations of 0°, 60°, and 120° (SE, segmented embryo; PE, projected embryo contour). (E) IoU validation for the reconstructed 3D model of embryo at different orientations. (F) Reconstructed 3D models of 9 different embryos showing irregularities in their individual shapes. (G) IoU validation for the reconstructed 3D model of different embryos.

For validation, we compared the projected images of the reconstructed 3D model of adult worms with the accurately segmented worm body in the bright-field images at corresponding orientations (Fig. 4A and Fig. S21C). The most commonly used metric for evaluating 3D reconstruction performance is the reprojection intersection over union (IoU) [48,49], which denotes the ratio between the projected images of the reconstructed 3D model and the segmented worm body in the bright-field images at corresponding orientations. A higher IoU indicates a better fit between the projected images of the reconstructed 3D model and the original worm body in bright-field images. To validate the accuracy of the reconstructed 3D model more comprehensively, the IoU was first calculated to assess the variance of the same 3D model from different viewpoints, ranging from 0° to 180° with an increment of 30° (Fig. S21). Importantly, IoU values at different orientations are higher than 95%, and thus, the average IoU value of our reconstructed 3D model was calculated to be 96.73% ± 0.41% (*N = 12*) (Fig. 4B), much higher than the recently reported IoU of 73.4% in the 3D reconstruction of objects via other methods [50]. This result demonstrates that the reconstructed 3D model maintains high precision at different viewpoints. Next, we applied our algorithms to the 3D reconstruction of different adult worms, and the average IoU values for the 3D reconstruction models of different adult worms were calculated to be 95.76% ± 1.05% (Fig. 4C), which further demonstrates that the high efficiency and accuracy achieved by the proposed algorithms for 3D reconstruction is reproducible.

In contrast to the rod-like shape of adult worms, a *C. elegans* embryo has an oval shape. Given that an embryo has a diameter of ~30 μm and a length of ~50 μm, 2D bright-field images of rotated embryos were captured under a 20× objective using conventional microscopy. Due to the background noise and low contrast between the embryo and background [Fig. S23A(i)], accurate segmentation of embryos in the original images was not possible (Fig. S15). We thus used the customized algorithms designed for the 3D model reconstruction of adult worms with some minor changes to reconstruct the 2D images into a 3D model of the embryo (see the Supplementary Materials). The reconstruction of the embryo follows the processing sequence of image super-resolution [Fig. S23A(ii)], embryo detection and segmentation [Fig. S23A(iii)], accurate measurement of rotation period, in-plane alignment of the detected embryo [Fig. S23A(iv) and A(v)], and 3D reconstruction (Figs. S22 and S23).

For validation, we compared the projected images of the reconstructed 3D model of the embryo with the accurately segmented embryo in the bright-field images at the corresponding orientations (Fig. 4D and Fig. S23C). To validate the accuracy of the reconstructed 3D model more comprehensively, the IoU was once again calculated to assess the variance of the same 3D model from different orientations, ranging from 0° to 180° with an increment of 30° (Fig. S23). Similar to our data with adult worms, most of the IoU values at different orientations are higher than 95% and the average IoU value of the reconstructed 3D model was calculated to be 96.29% ± 1.60% (Fig. 4E), which is sufficiently high to demonstrate that the reconstructed 3D model maintains high precision at different viewpoints [50]. Finally, we applied the proposed algorithms to the 3D reconstruction of different embryos at gastrulation stage and identified remarkable morphological differences between each embryo (Fig. 4F). The average IoU values for the reconstructed 3D models of different embryos were calculated to be 95.14% ± 0.75% (*N = 12*) (Fig. 4G), which further demonstrates high efficiency and accuracy of our algorithms in the 3D reconstruction of embryos when used in conjunction with the developed robotic system.

### 3D morphological phenotyping of RNA binding protein mutants

RNA binding proteins play an important role in regulating all aspects of RNA metabolism, and these proteins are associated with human health and disease [51,52]. Recently, to shed light on the role of RNA binding protein genes in animal development and physiology, we have knocked out specific RNA binding protein encoding genes in *C. elegans* and conducted systematic single and double mutant phenotyping of these strains using competitive fitness assays of moderate throughput [52]. However, to get a better understanding of how individual RNA binding proteins and the synthetic effect of RNA binding proteins impact animal development and physiology, additional phenotypic characterization of quantitative traits will complement these efforts. Given our development of a platform for 3D reconstruction of worms at different developmental stages, we explored its potential for 3D morphological phenotyping of RNA binding protein mutants by quantifying 5 key morphological metrics, which are the surface area, volume, length, maximum width, and the ratio of the length to the maximum width, of synchronized embryos at gastrulation stage and young adult worms.

**Fig. 5.**
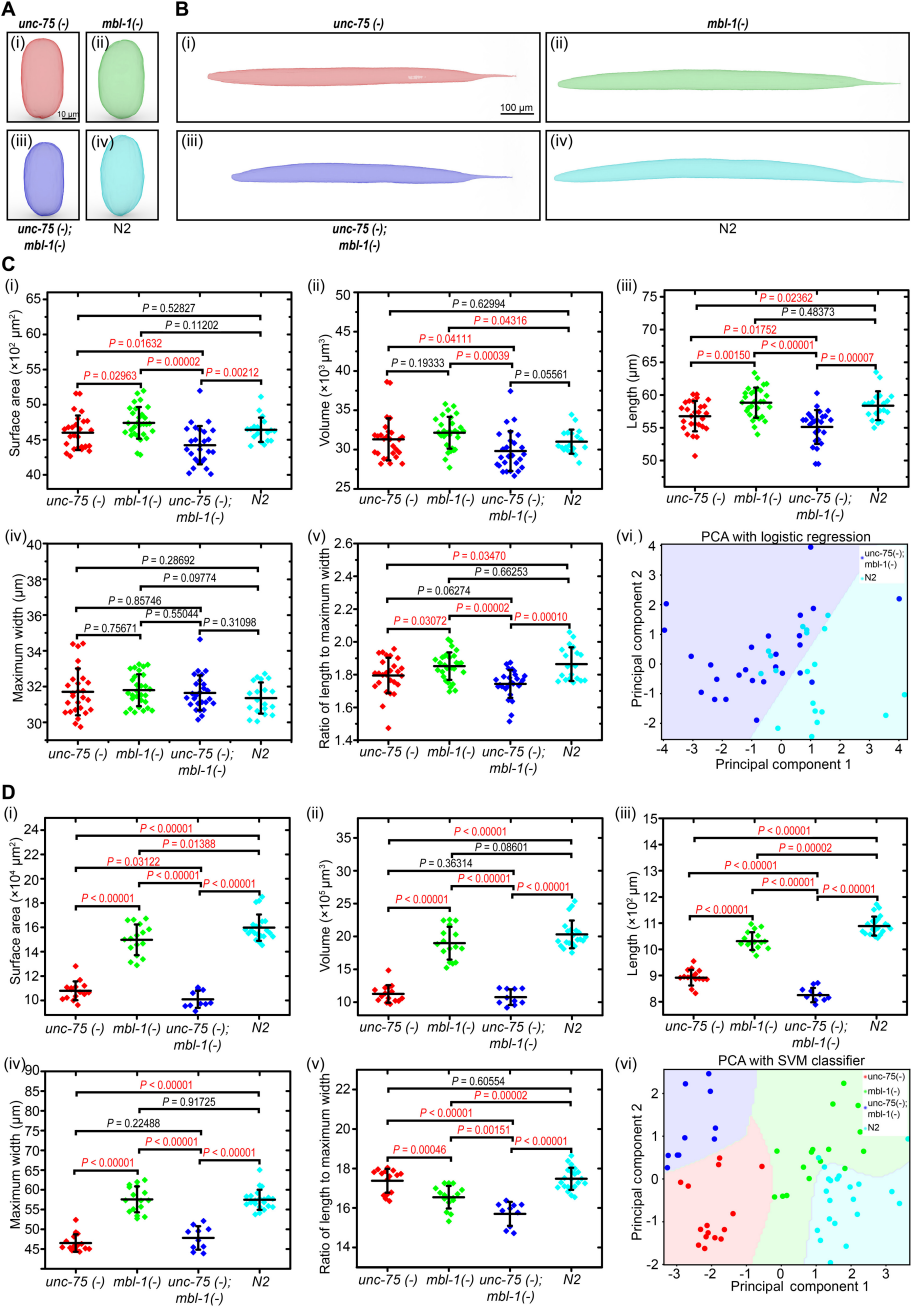
Reconstructed 3D models and 3D morphological phenotyping of the RNA binding protein mutants and N2. (A) Reconstructed 3D models of embryos at gastrulation stage from RNA binding protein mutants and N2: (i) single mutant: JAC624 [*unc-75(csb20)*], (ii) single mutant: JAC634 [*mbl-1(csb30)*], (iii) double mutant: JAC984 [*unc-75;mbl-1*], and (iv) N2. (B) Reconstructed 3D models of young adults from RNA binding protein mutants and N2: (i) single mutant: JAC624 [*unc-75(csb20)*], (ii) single mutant: JAC634 [*mbl-1(csb30)*], (iii) double mutant: JAC984 [*unc-75;mbl-1*], and (iv) N2. (C) Quantification of 5 key morphological metrics of embryos from different strains: (i) surface area measurement, (ii) volume measurement, (iii) length measurement, (iv) maximum width measurement, (v) ratio of length to maximum width, and (vi) logistic regression for the classification of the double mutant JAC984 [*unc-75;mbl-1*] and N2. (D) Quantification of 5 key morphological metrics of young adults from different strains: (i) surface area measurement, (ii) volume measurement, (iii) length measurement, (iv) maximum width measurement, (v) ratio of length to maximum width, and (vi) support vector machine (SVM) classifier for the classification of all strain.

As a proof of concept, we focused on a specific pair of conserved RNA binding proteins, UNC-75/CELF and MBL-1/MBNL, which have been identified to synergistically regulate neurite development at larval stages (S.P. and J.A.C., unpublished results). We screened *unc-75* and *mbl-1* single mutant animals as well as double mutants for morphological phenotypes from 3D models of mutants and N2 wild-type worms (representative models in Fig. 5A and B). Outlines of transverse section of young adults and embryos at selected positions were displayed in Fig. S24, showing the different morphologies of young adults and embryos from different strains.

Compared to wild-type embryos*, unc-75(csb20)* mutant embryos showed significant differences in the length [*P* = 0.02362, Fig. 5C(iii)] and the ratio of length to maximum width [*P* = 0.03470, Fig. 5C(iv)], while there were no significant differences in the surface area [*P* = 0.52827, Fig. 5C(i)], volume [*P* = 0.62994, Fig. 5C(ii)], and maximum width [*P* = 0.28692, Fig. 5C(iv)]. In contrast, *mbl-1(csb30)* mutants have a relatively larger volume [*P* = 0.04316, Fig. 5C(ii)] but no significant differences in the surface area [*P* = 0.11202, Fig. 5C(i)], length [*P* = 0.48373, Fig. 5C(iii)], maximum width [*P* = 0.09774, Fig. 5C(iv)], and the ratio of length to maximum width [*P* = 0.66253, Fig. 5C(v)] of embryos when compared to wild type. These results indicate that the loss of these individual RNA binding proteins has distinct effects on morphological parameters of embryos when compared with wild-type animals.

Typically, when double mutant phenotypes can be explained by the additive effects of the corresponding single mutant phenotypes, it is generally assumed that the corresponding genes being studied function independently of each other [53]. However, when double mutant phenotypes deviate from this expected additive effect, this phenomenon is defined as a genetic interaction, which can reveal functional relationships between genes [52]. Interestingly, we identified that *mbl-1* and *unc-75* genetically interact to determine several embryo size and shape properties. The surface area, which was significantly lower in double mutants compared with wild type [*P* = 0.00212, Fig. 5C(i)], showed no significant difference between the wild type and single mutants [*unc-75(csb20)*: *P* = 0.52827; *mbl-1(csb30)*: *P* = 0.11202, Fig. 5C(i)]. In addition, compared to the *unc-75;mbl-1* double mutants, a significant decrease in the surface area of the *unc-75* single mutants [*P* = 0.01632, Fig. 5C(i)] and the *mbl-1* single mutants [*P* = 0.00002, Fig. 5C(i)] existed. Given that the significant decrease in the surface area was observed in double mutants, which was not present in either single mutant, a genetic interaction between *mbl-1* and *unc-75* in shaping the surface area of embryo was identified. Moreover, the length, which was significantly lower in double mutants compared with wild type [*P* = 0.00007, Fig. 5C(iii)], was also lower in double mutants compared to *unc-75* single mutants [*P* = 0.01752, Fig. 5C(iii)]. However, the exacerbation of this phenotype in the double mutant cannot be explained by additivity, because *mbl-1* single mutants and wild-type animals show similar profiles of this metric [*P* = 0.48373, Fig. 5C(iii)], which implies that there exists a genetic interaction between *mbl-1* and *unc-75* in the length of embryo.

We similarly compared morphological measurements of young adults (Fig. 5D). Compared to wild-type young adults, *unc-75(csb20)* mutants show significant differences in the surface area [*P* < 0.00001, Fig. 5D(i)], volume [*P* < 0.00001, Fig. 5D(ii)], length [*P* < 0.00001, Fig. 5D(iii)], and maximum width [*P* < 0.00001, Fig. 5D(iv)], while there was no significant difference in the ratio of length to maximum width [*P* = 0.60554, Fig. 5D(v)]. In contrast, *mbl-1(csb30)* mutants have a relatively smaller surface area [*P* = 0.01388, Fig. 5D(i)], length [*P* = 0.00002, Fig. 5D(iii)], and the ratio of length to maximum width [*P* = 0.00002, Fig. 5D(v)], but no significant differences in volume [Fig. 5D(ii)] and maximum width [Fig. 5D(iv)] compared to wild-type adults. Finally, the simultaneous loss of *unc-75* and *mbl-1* led to significant decrease in surface area [*P* < 0.00001, Fig. 5D(i)], volume [*P* < 0.00001, Fig. 5D(ii)], length [*P* < 0.00001, Fig. 5D(iii)], and maximum width [*P* < 0.000012, Fig. 5D(iv)] compared to wild type. Interestingly, we again identified a genetic interaction between *mbl-1* and *unc-75* in our adult measurements. The ratio of length to maximum width, which was significantly lower in double mutants compared with wild type [*P* < 0.00001, Fig. 5D(v)], was also lower in double mutants compared to *mbl-1* single mutants [*P* = 0.00151, Fig. 5D(v)]. However, the exacerbation of this phenotype in the double mutant cannot be explained by additivity, because *unc-75* single mutants and wild-type animals show similar profiles of this metric [*P* = 0.60554, Fig. 5D(v)].

We next investigated whether the 3D morphological phenotype differences could be exploited for the reliable discrimination between the embryos and adults from the mutants and the wild type. The correlation between different morphological metrics of animals from different strains was plotted (Figs. S25 to S28), indicating that there were substantial overlaps within each morphological metric of embryos from different strains, while young adults from different strains can be easily distinguished. After performing logistic regression on the principal components analysis (PCA) [Fig. 5C(vi) and Fig. S29], we found that only the embryos of double mutants can be effectively distinguished from those of wild type through the condensed 3D morphological phenotype information represented by the principal components. In contrast, we found that young adults from each strain can be perfectly distinguished from each other through this latter approach [Fig. 5D(vi) and Fig. S29]. As shown in Fig. 5D(vi), 60 of 62 samples lay in the correct region.

Taken together, these results demonstrate that our 3D morphological screening approach provides additional quantitative phenotypic information and can also identify genetic interactions among mutant strains in which highly conserved RNA binding proteins are mutated, deepening understanding of disease mechanisms and providing avenues for the potential therapeutic targets.

## Discussion

In this study, we have developed a robust robotic system that enables the stable, uniform, and consistent rotation of *C. elegans* for clear and comprehensive observation of features via multi-view fluorescent imaging and quantification of morphological characteristics via precise reconstruction of 3D models. Specifically, by moving *C. elegans* rightward at a relatively small constant speed, the frictional force and hydrodynamic forces that are experienced by *C. elegans* are sufficiently gentle, allowing for the stable and consistent rotation of worms in a slow fashion while ensuring that rotated animals remain in focus.

In contrast to other rotation methods that require specific substrate designs, complicated device fabrication, expensive equipment, and elaborate operation procedures, our worm rotation method is simple, reliable, and independent of the substrate, making it easily applicable and highly advantageous in many applications, especially those necessitating a thin coverslip as the sample substrate for high-resolution imaging. Furthermore, our rotation method offers additional outstanding advantages. A rotational torque induced by combination of frictional force and hydrodynamic forces allows for contactless, precise, consistent, and stable rotation of *C. elegans*. This rotation mechanism is independent of the worm body shape and size. With its contactless nature and independence of the body shape/size, the proposed rotation method enables stable rotation of worms across different developmental stages and various mutant strains with varying body shapes/sizes. The slow rotation offers superior control over worm manipulation and provides the advantage of capturing a sufficient sequence of 2D images within a full rotation, enabling precise 3D reconstruction of worms. In addition, since there exists a consistent gap between the worm body and the open ends of microfluidic device during the rotation process, the entire worm body can be clearly observed. Finally, the movable microfluidic device and the capability of simultaneous manipulation of multiple samples allows for the rotation of animals in a successive fashion.

We demonstrated the utility of our system for imaging fluorescently labeled cell types and neurites, overcoming some of the limitations of widefield and confocal microscopy through the controlled rotation of worms. We also created, for the first time, the precise 3D reconstruction of *C. elegans* at embryonic and adulthood stages of development. Relying on the reconstructed 3D models, we were able to accurately quantify the change in the 3D morphological phenotyping of mutant strains. Through the precise measurement of morphological changes, we discovered that the loss of RNA binding proteins had distinct effects on different morphological metrics of embryos and young adults, and further characterized the genetic interaction between *unc-75* and *mbl-1*, which encode conserved RNA binding proteins with orthologs implicated in neurological and muscular disorders [54]. UNC-75 is broadly expressed in the nervous system, while MBL-1 is expressed in the nervous system and in other tissues [55–57]. Body size in *C. elegans* is largely controlled by the transforming growth factor-β (TGF-β) signaling pathway, which responds to environmental signals through the nervous system, including chemosensory and dopaminergic neurons [58,59]. It will be interesting to determine if the genetic interaction we observe between *unc-75* and *mbl-1* is a result of aberrant function in the nervous system or an interplay between neurons and other tissues.

More broadly, using our rotation method and 3D reconstruction algorithms, researchers could envision performing comprehensive fluorescent imaging of complex cell morphologies or 3D morphological phenotyping of larger numbers of transgenic strains (Fig. S30), taking advantage of our highly effective worm rotation system and the growing toolbox of available transgenesis approaches in *C. elegans* [60]. Moreover, since morphology and body size are quantitative traits, our platform could be used to survey how natural genetic variation can contribute to body size evolution, taking advantage of available strain collections such as the *Caenorhabditis* Natural Diversity Resource (CaeNDR) [61]. In addition to *C. elegans*, we also anticipate that this robotic system can be adapted to the smooth rotation of cells, *Drosophila* larvae, zebrafish larvae, and other small organisms. Taken together, our robotic system will open the door to a wide range of applications including genetic screening, drug screening, and fundamental discovery in development, neuroscience, and disease research.

## Materials and Methods

### Strain preparation

Both wild-type and transgenic worms used in this study were cultured on new nematode growth media (NGM) plates seeded with the bacterial food *Escherichia coli* OP50-1at 21 °C. Worm strains used were N2 Bristol (wild-type), TG2435 *vtIs1* [*dat-1p::GFP + rol-6(su1006)*] *V*, JAC769 *csbIs30* (*Pift-20::EBFP2 + Pacr-5::EBFP2 + Peat-4::CyOFP1 + Punc-17::mScarlet + Prgef-1::mNeptune2.5*), RW1596 *stEx30* [*myo-3p::GFP::myo-3 + rol-6(su1006)*], NC1686 *wdIs51* [*F49H12.4::GFP + unc-119(+)*], JAC624 *unc-75*(*csb20) I*, JAC634 *mbl-1*(*csb30) X*, JAC984 *unc-75(csb20) I; mbl-1(csb30) X* . Before the rotation process, worms at larval and adulthood stages were suspended in levamisole solution at a concentration of 0.6 mg ml^−1^ for 10 to 30 min to ensure that all animals were anesthetized. Synchronized young adult nematodes for the phenotypic analysis were obtained using the following protocol. Briefly, 3 adult worms from different strains were first transferred to new NGM plates and then cultured at 21 °C for 3 to 4 d. After that, the young adult worms, which have a few embryos inside the gonad, were selected for 3D morphological phenotyping. The synchronous populations of embryos at gastrulation stage were obtained by cutting anesthetized young adult worms with a needle (27-gauge × 1 ^1^/_4_″ to 0.4 × 32 mm, Nipro) in the custom-made reservoir, which was filled with M9 buffer.

### System setup

All components integrated into the robotic system were interfaced with a computer workstation (Dell Precision 7820, 8-Core 3.0 GHz CPU) with a data acquisition card (model 826, Sensoray). These components consist of an inverted microscope (Olympus IX83), a motorized micromanipulator (MX7600R), a digital camera (scA640-74fc, Basler), and a pressure regulator. The custom-made reservoir, which was filled with M9 buffer, was held by the motorized XY stage (Prior, Precision III). A custom-designed C++ program was developed to control the components of the robotic system and to acquire images. Artificial intelligence-based superresolution and segmentation was trained on the GPU (RTX A6000, NVIDIA) using the deep learning frameworks of Pytorch and PaddlePaddle.

### Statistical analysis

No statistical methods were used to predetermine the sample sizes. Statistical analyses were done afterward without interim data analysis. Two-tailed Student’s *t* test was performed for comparison between 2 groups of samples. All data were collected and processed randomly. All data were expressed as means ± SD. *P* value less than 0.05 was considered statistically significant.

## Data Availability

All of the experiment data in this work are presented in the main text and the Supplementary Materials. All original code and model weights used for the 3D reconstruction of *C. elegans* are available at https://github.com/MBL-Group/WoRecons.

## Supplementary Materials

Supplementary 1 Supporting Text Figs. S1 to S30 Table S1 Movies S1 to S10 References
