# Supplementary material for: High-Resolution Imaging and Morphological Phenotyping of C. elegans through Stable Robotic Sample Rotation and Artificial Intelligence-Based 3-Dimensional Reconstruction
Source: Research (Wash D C). 2024 Oct 30;7:0513. doi: 10.34133/research.0513 (PMC11522223; doi:10.34133/research.0513)
Supplement: Supplementary 1 — Supporting Text Figs. S1 to S30 Table S1 Movies S1 to S10 References [file research.0513.f1.zip › Support Information-RESEARCH-D-24-01072-Clean Version-Updated.docx]

**Supporting Information**

**High-resolution imaging and morphological phenotyping of *C. elegans* through stable robotic rotation and AI-based 3D reconstruction**

Peng Pan^1†^, Pengsong Zhang^1†^, Sharanja Premachandran^2^, Ran Peng^3^, Shaojia Wang^1^, Qigao Fan^4^, Yu Sun^1,5^, John A. Calarco^2*^, Xinyu Liu^1,5*^

^1^Department of Mechanical and Industrial Engineering, University of Toronto, Toronto, Ontario M5S 3G8, Canada

^2^Department of Cell & Systems Biology, University of Toronto, 25 Harbord St, Toronto, ON M5S 3G5, Canada

^3^College of Marine Engineering, Dalian Maritime University, Dalian 116026, China

^4^School of Internet of Things Engineering, Jiangnan University, Wuxi, 214122, China

^5^Institute of Biomedical Engineering, University of Toronto, 164 College Street, Toronto, Ontario, M5S 3G9, Canada

* Address correspondence to: [john.calarco@utoronto.ca](mailto:john.calarco@utoronto.ca); [xinyu.liu@utoronto.ca](mailto:xinyu.liu@utoronto.ca)

†These authors contributed equally to this work.

**This PDF file includes:**

Supporting text

Figures S1 to S30

Tables S1

Legends for Movies S1 to S10

SI References

**Other supporting materials for this manuscript include the following:**

Movies S1 to S10

Supporting Information Text

**Microfluidic device fabrication**

The microfluidic device was integrated into the robotic system, serving as an important part for the stable and controlled rotation of worms at different developmental stages. The microfluidic device, comprised of two PDMS layers, was fabricated through the multilayer soft lithography. First, a SU-8 (2075, Microchem) mold of the top PDMS layer with a thickness of 25 μm was achieved by the standard lithography [**Fig. S2B(i)**]. Subsequently, a precursor mixture comprising a PDMS base and a cross-linker (Sylgard 184, Dow Corning) at the w/w ratio of 5:1 was first poured onto the SU-8 mold, then cured at 80 °C for 45 min. After that, a thin PDMS layer with a thickness of 14 μm was obtained by spin-coating the precursor mixture of PDMS base and cross-linker (Sylgard 184, Dow Corning) at the w/w ratio of 10:1 onto a silicon wafer, and then cured at 80 °C for 45 min [**Fig. S2B(ii)**]. In the following, the top PDMS player was peeled off from the SU-8 mold and bonded to the 14 μm-thick PDMS layer following the puncturing of inlet holes **[Fig. S2B(iii)**]. After secure bonding, the PDMS device was peeled off from the silicon wafer [**Fig. S2B(iv)**]. Note that the microfluidic device was mounted with a tilting angle of 30° on the right micromanipulator. To avoid any image shading of the worm body by the device edge in the entire process of worm rotation, a smooth slope of 45° was then cut at the side wall of the microfluidic device where the open ends of the microchannels are arranged [**Fig. S2C**]. Subsequently, the bottom side of the microfluidic device, except for a small area near the open ends, was bonded to a flat and transparency film (thickness: 0.1 mm, Inkjet Transparency Film, Staples) which served as a supporting layer by using a double-sided tape with thickness of 25 μm (8171CL, 3M) [**Fig. S2, B(v) and B(vi)**]. This prevented the bottom PDMS layer from adhering to the upper side of the microchannels due to the deformation under the action of negative pressure, thus avoiding blockage of the microchannels. Finally, the microfluidic device was bonded to a pre-cut cast acrylic sheet (PMMA) plate (thickness: 1/16″, McMaster-Carr) by using a double-sided tape, providing secure fixation onto the right micromanipulator [**Fig. S2, B(vii) and B(viii)**]. The exploded view of microfluidic device is shown in [**Fig. S2D**].

**Estimation of the flow rate inside microchannel**

It was observed that the medium inside the microfluidic channels slowly flowed out of the microchannel open ends under the action of gravity once the negative pressure was no longer applied by turning off the miniaturized pressure regulator. To estimate the flow rate inside the microchannel, the movement of a microparticle which was brought outside the microchannel by the flow was observed. As shown in **Fig. S3,** the microparticle which was transported outside the microchannel by the flow moved leftward with a displacement of 63.87 μm in 0.33s. Thus, the flow rate was estimated to be ~191.23μm/s, which was used for the simulation analysis thereafter.

**Numerical simulation analysis**

To investigate the rotation of *C. elegans*, a numerical simulation analysis was performed. The rotation of *C. elegans* was simplified by two-dimensional (2D) simulation domain [**Fig. S4**] and simulated by COMSOL Multiphysics by using the fluid-solid interaction module in which Laminar Flow and solid mechanics as well as moving mesh modules were included (COMSOL Inc., Version 6.0). In the 2D simulation domain, a reservoir was connected to a microchannel and *C. elegans* was located 50 µm away from the open ends of the microchannel. The reason why 50 µm was chosen was that there exists a consistent gap between the rotated worm and the open-end edge of microfluidic device during the rotation process, and the consistent gap between the rotated worm and the open ends of the microchannel was close to 50 µm [**Fig. 1**]. The size of the reservoir is listed as follows: AB=0.8mm, BC=1mm, and the CD boundary has a tilting angle of 75 degree. The 30-degree-tilted microchannel with a width of 25 µm was filled with solution and a flow velocity of 200 µm /s [**Fig. S3**] was applied to the inlet (inlet 1) of the microchannel. The motion of the moving substate BC in the horizontal direction was set to be 25 µm /s. As the solution in the reservoir moved with the substrate, the boundary AB was set to be the inlet 2 with the velocity of 25 µm /s. The diameter of *C. elegans* was set to be 50 µm.

The simulation is based on the Navier-Stokes equation, which is applied to solve the flow field in the model in which solution with dynamic viscosity and density of 0.001 Pa•s and 1000 kg/m3 was applied. The boundary condition at the *C. elegans* and the microchannel walls were set to 'no slip', assuming no relative fluid movement at the micro-gear sidewall. An “open boundary condition” was applied on the boundaries AD. A “slip wall” condition was applied on the substrate of BC. During the simulation, a moving mesh setup was applied to the computational domain to obtain satisfactory meshing and convergence quality. The torque induced by the frictional force was applied to the *C. elegans.* As there exists a consistent gap between the rotated worm and the open-end edge of microfluidic device during the rotation process, to simplify the model, *C. elegans* was fixed at the position which is 50 µm far away from the open ends of the microchannel. Finally, the time-dependent simulation analysis shows that the worm was rotated in an anti-clockwise direction (labelled by the line- “LR”), showing an anti-clockwise torque induced by the combination of frictional force and hydrodynamic forces was applied to the worm.

**Working principle of robotic rotation**

To achieve reliable rotation of worms at different developmental stages, we proposed a straightforward yet highly effective method utilizing the developed robotic system. First, the customized microfluidic device, mounted with a tilting angle of 30° on the right micromanipulator, was accurately positioned in the vertical position, ensuring the bottom edge at the open ends just touched the custom-made reservoir without any deformation [**Fig. 1, A and B**]. This would prevent the breakage of device and the formation of turbulent flow during the rotation process, due to the deformation of the microfluidic device. Later the mounted microfluidic device was connected with a plastic tube, and the plastic tube was then connected with the pressure regulator. The plastic tube, running from the inlet of the microfluidic device to its highest point [**Fig. S1C**], and the microfluidic channels were filled with M9 medium by activating the miniaturized pressure regulator. Once the negative pressure was no longer applied by turning off the miniaturized pressure regulator, the M9 medium inside the microfluidic channels and the tube would slowly flow out of the microchannel open ends under the action of gravity [**Fig. S3**]. The fluid flowing around the open ends of the microfluidic channels has a near-field region [**Fig. 1C**] *(62, 63)*. The near-filed region, having flow characteristics similar to those at the open ends of the microfluidic channels, is usually found within 0 < *x/D* < 6 (*x* represents the region length starting from the jet exit and *D* represents the equivalent diameter of the microfluidic channel) *(62, 63)*. As the Reynolds number within the microfluidic channel is lower than 0.33, the flow at the open ends of the microfluidic channel is laminar. Since the microchannels of the microfluidic device were designed to be 50 μm wide and 25 μm high, the equivalent diameter of each microfluidic channel is 33.3 μm. Thus, the near-field region is a laminar region, and the length of the laminar region is ~200 μm.

When a worm was brought to the open ends of the microfluidic device by moving the motorized stage of the microscope at a constant speed, it would be continually subjected to several forces at the beginning, including the frictional force (*F_pf_*) between the worm body and the substrate, and the drag force (*F_d1_*) as well as the lift force (*F_l1_*) resulting from the fluid flowing out of the open-ended channels [**Fig. 1C(ii)**]. The height of the microfluidic channel center at the open ends is 36.2 μm, which is greater than that of the worm body center (~25 μm). In combination with the 45° slope and the relatively greater height of the microfluidic channel center at the open ends, the hydrodynamic forces from the fluid flowing out of the microchannel open ends would mainly act on the top surface of the worm body [**Fig. 1D, Fig. S4**]. Since the hydrodynamic forces were mainly exerted on the top surface of the worm body while the frictional force acts on the bottom side of the worm body, a counter-clockwise torque (*T_1_*) would be thus exerted on the worm body. The closer the worm body is to the microchannel open ends, the greater the drag force would be. Due to the increasing drag force as the worm is moved close to the open-ended channels, the worm body would no longer move with the motorized stage, resulting in a relative motion between the worm and the solution in the reservoir that moved with the motorized stage. The relative motion would induce the drag force (*F_d2_*), the lift force (*F_l2_*) as well as the clockwise torque (*T_2_*) on the worm [**Fig. 1C(iii)]**. If the moving speed of the motorized stage is very slow, the rotation of the worm would be dominated by the frictional force (*F_pf_*) and the hydrodynamic forces (*F_d1_* and *F_l1_*) from the fluid flowing out of the open-ended channels while the effect of the hydrodynamic forces from the solution in the reservoir which moved with the motorized stage can be neglected. The drag force (*F_d1_*) would finally counterbalance the sum of the frictional force (*F_pf_*) and the drag force (*F_d2_*), preventing the worm body from contacting the open-ended channels of the microfluidic device. Eventually, a net counter-clockwise torque would be thus exerted on the worm body, resulting in the rotation of the worm [**Fig. S5**]. To ensure that worm rotation is achieved within the laminar region, the drag force (*F_d1_*) which increases with the maximum height of the plastic tube should not be excessive, allowing the worm to be brought close to the microchannel open ends while avoiding their contact with the worm. The experimentally determined maximum height of the plastic tube was 5 cm. Due to the small, constant speed of the motorized stage and the appropriate height of the plastic tube, the lift forces exerted on the worm body would also not be excessive, ensuring that rotated worms remain in focus. Thus, when coupled with microscopy, our proposed robotic system is amenable for the acquisition of high-quality images of animals at various perspectives.

**Rotation angle of the worm body by recognized patterns**

Rotation angle of worms can be monitored by visually recognizing their patterns. In particular, the cross-section through the middle portion of the anterior/posterior worm body at larval stages /adulthood mainly consists of three parts: the intestine, distal gonad, and proximal gonad *(21)*. In addition, the gonad roughly occupies half of the cross-section and the intestine takes up the remaining space. In brightfield mode, the intestine is mostly opaque, however, both the distal gonad and proximal gonad portions are transparent *(21)*. The rotation angle of worms at larval stages/adulthood can be monitored by visually recognizing the patterns of transparent gonads or opaque intestine [**Fig. S6**]. **Figure S6A(ii)** schematically shows the top view of worm at the orientation of 90°, indicated by the largest size of the observed transparent area under the bright field mode (or, conversely, indicated by the smallest size of the observed opaque area under the bright field mode). At the orientations of 180° and 270° [**Fig. S6, A(iii) and A(iv)**], the size of bright area reaches the minimum and maximum, respectively. In brightfield mode, the morphological pattern of the embryo/cell pattern inside the embryo varies at different orientations, as shown in **Fig. S6B**. The rotation angle of embryos can be monitored by visually recognizing the cell pattern inside embryos, in conjunction with the morphologies.

**Automatic alignment of the worm at the initial stage of worm rotation**

The microfluidic device is movable as it is mounted on the right micromanipulator. In addition, samples are loaded in the custom-made reservoir, which is held by the motorized stage of the microscope. When the initial position of a sample is away from the microfluidic device, it can be brought to the open ends of the microfluidic device by moving either the motorized stage or the right micromanipulator. Thus, there is no requirement for the initial in-plane position of the sample.

Before the rotation, worm usually is not aligned with the edge of the microfluidic device. Under the action of the frictional force and the hydrodynamic forces, worms with rod-like shape and oval shapes would be rotated around the Z-axis and finally aligned with the edge of the open-ended microfluidic device, as shown in **Fig. S8**.

Different from single cell which has sphere shape, *C. elegans* at embryonic stage, larval stages and adulthood have oval and rod-like shapes which make them very difficult to rotate around the Y-axis.

**Stoppage of worm rotation**

Once a worm was rotated to the desired orientation, the stoppage of worm rotation can be obtained by quickly moving the worm to the left, away from the open-ended channels of microfluidic device. As shown in **Fig. S9A**, the motorized stage started moving rightwards with a constant speed of 50 µm/s, and the worm was then brought close to the open ends of microfluidic device [**Fig. S9A**]. From **Fig. S9A** to **Fig. S9E**, the stable rotation of worm was observed. Once the worm was rotated to the 540 ° [**Fig. S9E**], the motorized stage started moving leftwards at a relatively large speed (250 µm/s) and the rotation of the worm was stably stopped with neglected orientation during this process as it was brought far away from the microchannel open ends [**Fig. S9F**]. The black arrow in each figure indicates the moving direction of motorized stage at each time point.

**Fluorescent Imaging of all head neurons in the anterior part of *C. elegans* (JAC769) from different orientations**

In addition to the clear imaging of specific neurons, we used the robotic system to image a strain (JAC769) expressing mNeptune2.5 (a far-red fluorescent protein) more broadly across the nervous system under the control of the *rgef-1* promoter *(21)*. This can demonstrate the compatibility of our robotic system with fluorescent imaging of all 302 neurons at distinct orientations using the widefield fluorescent microscopy. Upon rotation in 90° intervals, we easily obtained images of the left [**Fig. S10A**], ventral [**Fig. S10B**], right [**Fig. S10C**], and dorsal [**Fig. S10D**] perspectives of the same animal**.** At each rotation angle, we were able to visualize neurons not captured by other perspectives, highlighting the utility of controllable rotation. In particular, the ventral nerve cord was not captured in the fluorescent image [**Fig. S10D**] since it was rotated away from the objective which leads to the decrease in intensity and resolution of fluorescent signal. In each view, the white dotted ovals highlight the retrovesicular ganglion. The retrovesicular ganglion obtained at the 90° orientation was much clearer than those obtained from the other three orientations as the neurons were rotated close to the objective and they were separated from each other. The best imaging quality of neurons in the dotted rectangle was also observed at the 90° orientation in which both the quantity of observed neurons and the quality of fluorescent signal exhibited an increase.

**Incomplete fluorescent imaging of PVD dendrites by using confocal microscopy**

The incomplete 3D model reconstructed from the confocal imaging of the worm (NC1686) shows the signal of PVD dendrite which is away from the objective is missing when using the standard laser scanning confocal microscopy, as shown in **Fig. S10**. The results shown in **Fig. 2C** demonstrate that the developed rotation method enables clear and comprehensive imaging of PVD neurons and the bilateral PVD left and right (PVDL and PVDR) dendrites distributed over the majority of worm, providing a simple solution to overcoming limitations of confocal microscopy when imaging relatively thick tissue samples such as an adult *C. elegans*.

**Estimated volume of *C. elegans* based on single 2D image**

Conventionally, the volume of *C. elegans* was estimated by extracting the contour of *C. elegans* in a single 2D image and assuming that the cross-section of *C. elegans* was circular *(14)*. However due to the irregular shapes of *C. elegans* at different developmental stages, the estimated volume by employing the conventional method was inaccurate, as shown in **Fig. S12**. Based on the 2D images obtained at different orientations within a rotation cycle, there existed a significant variance between the maximum and minimum estimated volume of the same embryo, and the difference was calculated to be 33.17% by using the following equation, as shown in **Fig. S12A**. Also, the difference between the maximum and minimum volume of the same adult was calculated to be 18.63%, as shown in **Fig. S12B**. The huge variance between the estimated volume from different 2D images further demonstrated that the volume estimated by the conventional method was inaccurate which adversely affects the accuracy and consistency of genetic studies.

Difference between the estimated volume of the same embryo = 100% *(maximum estimated volume - minimum estimated volume) / minimum estimated volume.

**3D reconstruction workflow of *C. elegans* embryo and adult**

The 3D reconstruction workflow of *C. elegans* is divided into two major steps: (i) preprocessing of images and (ii) shape-from-silhouette based 3D reconstruction, as shown in **Fig. 3**. Typically, the low spatial resolution and the presence of background noise of the microscopy image poses challenges to the accurate segmentation of *C. elegans*. Thus, the animal in the original microscopy image will be segmented with irregular boundaries and edge sawtooth, preventing the precise analysis thereafter. To address these challenges, we developed a customized deep learning techniques (the section of “**AI for the super-resolution and denoising of images”** to be introduced below) that seamlessly integrate super-resolution and denoising strategies [**Fig. S13**]. The denoising component of the developed machine learning-based algorithms effectively mitigates the adverse effect induced by the background noise, thereby enhancing the overall quality of the acquired microscopy images. Simultaneously, the super-resolution process enhances the spatial resolution of original microscopy images, thereby making the morphological details of *C. elegans* clearer (the section of “**Super resolution and denoising of images”** to be introduced below). The combination of super-resolution and denoising strategies, inheriting advantages from both, provides a novel strategy for *C. elegans* segmentation with enhanced segmentation accuracy (the section of “**AI-based accurate segmentation of *C. elegans*”** to be introduced below). Subsequently, the template matching algorithm *(47)* was employed to measure the rotation speed of *C. elegans* (the section of “**Accurate measurement of *C. elegans* rotation speed”** to be introduced below)*.* As demonstrated in the main text, the rotation of the same adult worm and embryo are very uniform. Taking advantage of the uniform rotation and the template matching algorithm, we were able to calculate the rotational angle of *C. elegans* in each frame. During the worm rotation process, there existed small in-plane drift (yaw offsets) for the rotated worm. To create a precise 3D model, the segmented worm in each frame should be aligned (the section of “**Alignment of *C. elegans* body for precise 3D reconstruction”** to be introduced below). It should be noted that the tail of an adult worm is transparent and slender, which leads to the varying visibility at different orientations. Thus, at certain orientations, the segmented tail was incomplete. With the obtained frame featuring the longest tail within a rotation cycle, the tail completion for all frames within the same rotation cycle was performed which relies on the observed angle of the tail across frames. All in all, these preprocessing steps collectively facilitate precise reconstruction of 3D models of *C. elegans.*

Once the preprocessing steps were completed, aligned worms in each 2D image within a single rotation period were processed for the 3D reconstruction based on the customized shape-from-silhouette 3D reconstruction algorithms. In contrast to conventional estimation for adult worms, which often idealize cross-sections as perfect circles, a novel methodology was developed for the precise 3D reconstruction of adult worms.

First, the left contours of a rotated worm in each frame within a complete rotation cycle were obtained. In the following, these contours were coherently aligned along their central axes to form a unified 3D point cloud. Optimization was performed by utilizing the re-projection metric, Intersection over Union (IOU). Finally, the 3D reconstruction of adult worms with remarkable accuracy was generated by the developed algorithms (the section of “**Methodologies for 3D reconstruction of adults and embryos”** to be introduced below).

In contrast to the rod-like shape of adult worms, the *C. elegans* embryo has an oval shape with more irregular cross-sections. To create precise 3D model of the embryo, contour points of the rotated embryo at different orientations were first extracted. Later, the accurate 3D model of the embryo was reconstructed by aligning the contour points with the estimated rotation axis at each corresponding rotational angle (the section of “**Methodologies for 3D reconstruction of adults and embryos”** to be introduced below). The results finally demonstrate that the proposed 3D reconstruction methods are highly efficient and accurate as the 3D reconstruction of both adults and embryos yielded an IoU over 95% which surpassed the efficacy of other 3D reconstruction method *(50)*. Once the 3D reconstruction of adults and embryos were completed, post-processing steps were applied to the reconstructed 3D point clouds. A filtering method was employed to eliminate noise points resulting from segmentation errors. Subsequently, Poisson surface reconstruction techniques were harnessed to construct the surface of both adults and embryos. Furthermore, a texture mapping approach was implemented to enhance the realism of the 3D reconstructions. These postprocessing steps aimed to enhance the visual fidelity and accuracy of the reconstructed 3D models.

**AI for the super-resolution and denoising of images**

A Generative Adversarial Network (GAN)-based super-resolution method was employed to increase the quality of the original images. The architecture of the neural network utilized in this process is depicted in **Fig. S13**. This approach was inspired by the pioneering work of Chong Mou and colleagues *(64)*, specifically from the Metric Learning based Interactive Modulation for Real-World Super-Resolution (MM-RealSR) methodology. MM-RealSR's innovative strategy for interactive image restoration, rooted in metric learning and unsupervised degradation estimation, offered a compelling solution which is ideal for addressing the intricate challenges encountered in the domain of microscopy imaging. Usually, there exists many factors, including noise and blur, which significantly degrades the quality of microscopy images and affects their fidelity. To adapt to these intricacies, a deliberate shift from conventional Rectified Linear Unit (ReLU) activation functions to hard swish (hSwish) and Funnel activation (FReLU) was implemented. hSwish provides a smoother alternative to the ReLU, and FReLU introduces a spatially informed 2D activation function *(65).* Both hSwish and FReLU are good at capturing complex image features. This change would enhance the capability of models to discern intricate patterns and spatial nuances intrinsic to microscope images.

**Super resolution and denoising of images**

The model training was conducted on the NVIDIA A6000 platform. Throughout the training process, a dataset comprising 2,287 images captured under 20× magnification was assembled. A series of degradation operations were applied to these images, including a 4× down-sampling to generate low-resolution images for the training of super-resolution model. During the prediction step, 1.25× bilinear interpolation was applied to the original microscopy images captured under 4× magnification for upscaling. Subsequently, these upscaled images were fed into the model, and high-resolution images which are four times larger than that of original microscopy image were generated. **Fig. S14** shows the microscopy images of adult worms and embryos and resultant images processed by different methods including Bicubic interpolation, MM-RealSR, and our developed deep learning method. As shown in **Fig. S14**, the MM-RealSR method and our method provide noticeable improvements in image sharpness and quality. However, the MM-RealSR method would introduce excessive sharpness to the images, resulting in the distortion of surface textures [**Fig. S14A(iii)**], the loss of details [**Fig. S14B(iii)**], and the generation of artificial shape contours [**Fig. S14C(iii)**]. As shown in **Fig. S14,** our proposed method would better preserve the detailed information of *C. elegans* when compared to the MM-RealSR method. All in all, our proposed method not only enhances the image quality but also effectively preserve the details of *C. elegans* in microscopy images.

**AI-based accurate segmentation of *C. elegans***

The quality of segmentation is important for the precise reconstruction of 3D models thereafter. As depicted in **Fig. S15A** and **Fig. S15D**, segmentation of *C. elegans* in the original images utilizing the traditional OTSU threshold was achieved. However, the transparent regions of adult worms and embryos were not detected. Subsequently, a high quality of semantic segmentation approach, object-contextual representations for semantic segmentation (OCRNet) *(66)*, was employed to the worm segmentation in the original images. While this method provided significant improvements in the segmentation, it still suffered from the loss of details in the adult tail [**Fig. S15B]** and the noticeable aliasing artifacts of detected contours [**Fig. S15E]**. As mentioned above, the high-resolution images were obtained by employing deep learning algorithms. Later, the semantic segmentation approach (OCRNet) was employed to the worm segmentation in the high-resolution images generated by our methods. As shown in **Fig. S15C and Fig. S15F**, the segmentation of adults and embryo were perfect with smooth detection of contours and accurate detection of tails. Finally, the OCRNet method was employed to the *C. elegans* segmentation thereafter. The training process of semantic segmentation of adults and embryos were shown in **Fig. S16**.

**Accurate measurement of *C. elegans* rotation speed**

The accurate orientation estimation of imaged objects is of paramount significance for the precise 3D reconstruction. However due to the transparent body of *C. elegans*, orientation estimation is challenging when using the conventional feature detection and matching methods as they are susceptible to errors when dealing with transparent objects. To accurately measure the rotation cycle of the *C. elegans*, we developed a new method for the orientation estimation which was inspired by the concept of loop closure detection commonly applied in Simultaneous Localization and Mapping (SLAM). The method starts by converting the captured video into a sequence of images, followed by super-resolution processing of each frame. Subsequently, we employ a semantic segmentation technique to accurately extract adult worms and embryos from the background, reducing the effect of background noise on the measurement of rotation speed. In the following, a template matching method was employed to calculate the similarity between each frame and the initial frame, enabling the accurate measurement of a complete rotation cycle. As shown in **Fig. S17A**, the first frame of the video of adult rotation was selected as the reference frame, and template matching algorithm was performed on the subsequent frames. Later the highest similarity score was obtained for the frame of 252, which was considered as the last frame of the first rotation cycle and the first frame of the second rotation cycle. In the following, the frame of 500 was selected as the last frame of the second rotation cycle. The small variances between the measured rotation cycles of young adult worms/embryo [**Fig. S17, B and C**] further demonstrated that the rotation of *C. elegans* was uniform. In combination of the uniform rotation and accurate measurement of each rotation cycle, the rotation angle for each frame within a complete rotation cycle was calculated by dividing 360 degrees by the total number of frames in the rotation cycle, which will be then utilized for the precise 3D reconstruction thereafter. The template matching algorithm was also employed to identify the image within the second rotation cycle which has the highest similarity score compared to the rotated worm at a certain orientation within the first rotation cycle [**Fig. S17A**]. The frame number of adult at identical orientations within the first and second rotation cycle was almost same, which further demonstrated that the rotation of *C. elegans* was uniform [**Fig. S17A**].

**Alignment of *C. elegans* body for precise 3D reconstruction**

Due to the irregular shapes of *C. elegans* at different developmental stages as well as the state of free movement, there exists small yaw offsets in the plane during the rotation process. The misalignment of rotated worms in each frame would lead to inaccurate 3D reconstruction of adults and embryos thereafter. It should be noted that the tail of an adult worm is transparent and slender, which leads to the varying visibility at different orientations. Thus, at certain orientations, the segmented tail was incomplete. Here, we presented novel methods for the alignment of worm body and the tail completion, which facilitates the precise reconstruction of adult worms and embryos for the accurate phenotypic analysis thereafter. The detailed information of the method for the correction of yaw offset was described in **Table S1**. First, the center point [labelled as *o* in **Fig. S18A(i)**] of accurately segmented worm body was calculated. Based on the calculated center point, two dash lines which were parallel to the *x*-axis were drawn, one of which was above the center point [labelled as *line 1* in **Fig. S18A(i)**] and the other was below the center point [labelled as *line 2* in **Fig. S18A(i)**]. The distance between the top line and the center point was one-fourth of the length of worm. Also, the distance from the bottom line and the center point was one-fourth of the length of worm. As shown in **Fig. S18**, there existed four intersection points (points *a, b, c, d*) between the two parallel lines and the contours of worm. Later, two straight lines (*ab* and *cd*) were formed by connecting two intersection points on the left side and two intersection points, respectively. The angle for the yaw offset correction was then calculated based on the two lines and *y*-axis, as shown in **Table S1**. Finally, the adult worm was aligned based on the corrected angle, as shown in **Fig. S18**, for the precise 3D reconstruction thereafter. In some frames, we found the tail of an adult worm was incomplete. To perform the tail completion, the maximum tail length within a complete rotation cycle was measured. Subsequently, in each frame, the tail's angle was computed, and then the tail was completed in combination of the computed angle of tail and the measured maximum length of tail [**Fig. S18A]**. The method was also employed to successfully align the rotated embryo at different orientations with minor change of distance between the center point and the bottom/top dashed lines [**Fig. S18B]**.

Given that both *C. elegans* adults and embryos have non-uniform shapes and mass distributions, achieving pure rolling during rotation is indeed challenging. This irregularity inevitably leads to minor yaw angle shifts around the *Z*-axis, as well as slight positional shifts in the *X* and *Y* directions. To address the minor positional shifts that occur during rotation, we applied image processing techniques to correct these deviations, facilitating the subsequent 3D reconstruction process.

For adults, specifically, after angle correction, we observed that the tail of the adult *C. elegans* is very thin and sometimes unclear, leading to inconsistent segmentation results for the tail. However, the head region is more distinct and thus yields better segmentation results, making it more suitable for positional alignment. In combination with alignment of the recognized head and the centroid of segmented adult worm, the positional shift of rotated worm can be corrected.

For embryos, positional alignment after angle correction is also necessary to ensure consistency of the 3D reconstruction. Since the length of the embryo is same, alignment of the centroid point of the segmented embryo contour in each microscopy image allows for correction of the positional shift along its body length. As the width of the embryo at each orientation is not consistent, it can cause shifts in the rotation axis. Therefore, it is crucial to extract the rotation axis of the rotated embryo in each microscopy image. As shown in **Fig. S19**, specifically, during the rotation of the embryo, the orientations when a contour is either in contact with the substrate or clearly observed from the top view in which its width can be clearly measured always differ by 90 degrees. Thus, the embryo can be equivalent to being rotated on a virtual substrate on the left side [**Fig. S19**]. To calculate the rotation axis, we align the embryo within the microscopy image to the left [the left is indicated by the virtual substrate after perspective switching in **Fig. S19**], allowing for precise calculation of the rotation axis position during the rotation cycle. Finally, in combination with the calculated rotation axis and the alignment of embryo contours, precise 3D reconstruction of embryo can be obtained.

These image processing methods effectively reduce or eliminate the impact of rotational and positional deviations due to the non-uniform shape and mass distribution, thereby improving the accuracy of the 3D reconstruction.

**Methodologies for 3D reconstruction of adults and embryos**

Embryos and adults, which are the earliest and latest stages of *C. elegans,* respectively, have distinct shapes. In contrast to the rod-like shape of adult worms, a *C. elegans* embryo has an oval shape. In addition, embryos and adults are the two stages which are widely employed in the biological studies. This is because the *C. elegans* embryo is an excellent model system to study biological mechanisms such as cellular processes and developmental regulation at a very early stage. As adult worms have fully developed nervous system and structures, comprehensive studies can be achieved by using the worms at adulthood stage. Thus, in the following, 3D models of both adults and embryos were reconstructed based on the preprocessing steps mentioned above. As the embryos and adults have distinct shapes, the methods developed for the reconstruction of embryos and adults differ slightly.

For adults, the contour width at each point of the left contour was obtained by calculating the distance between the contour point of the left contour and the pairwise point of the right contour. Half of contour width at each point of the left contour was then achieved [Labelled by the short lines in **Fig. S20A**]. In the subsequent process, the center point between each left contour point and its pairwise right contour point was aligned with a central axis (an axis parallel to the *y*-axis) after which all the left contour points were kept and all the right contour points were removed. Later, the left contours of rotated worm in each frame within a complete rotation cycle were obtained. In combination with the accurate measurement of rotational angle of worm in each frame, a preliminary 3D point cloud of the worm was achieved by the alignment of the central axis in each frame and the coordinate transformation of the left contours obtained in each frame. Subsequently, the preliminary 3D point cloud was corrected as follows. The left contour information of the rotated cell in the two original microscopy images, where the cell exhibits a 90 ° angle difference between the two images [**Fig. S20B**], were extracted for the position correction of each cross-section of the preliminary 3D point cloud. To validate if the corrected 3D point cloud was accurate, average intersection over union (IoU) between the projected images of the corrected 3D model is always employed as reprojection metric to evaluate the accuracy of reconstructed models *(50,67,68)*. Therefore, the accurately segmented adults in the bright-field images at different orientations were calculated. Later, the two original microscopy images, in which the rotation angle between the worms were 90 °, was changed for the correction of the preliminary 3D point cloud, and the average IoU was calculated again [**Fig. S20B**]. Before computing the IoU between the reprojected 3D model image and the original image, a statistical filtering algorithm was employed, which served the purpose of mitigating the presence of discrete point clouds while concurrently diminishing the extent of noise in the data *(69)*. Finally, the 3D point cloud which provided the highest IoU would be stored and chosen as the final 3D model of the adult.

For the embryos, due to the smaller aspect ratio and clearer contour information in the microscopy images obtained under 20 × objective, the original contour information was directly used for the precise 3D reconstruction of embryos. Since embryos have irregular shapes and remain in contact with the substrate during the rotation process, the rotation axis changes in real time. Here we made an alignment between the left half contours of the embryo [**Fig. S22A**], in which the embryo was effectively equivalent to being rotated on a substrate on the left side for the precise 3D reconstruction thereafter. The distance between the real-time rotation axis and left substrate was half of the contour width in each frame. To realize the 3D reconstruction of embryos, the contour information was first extracted [**Fig. S22A**], and the rotation axis was estimated based on the measured width in each frame. In combination with the estimated rotation axis and the accurate measurement of rotational angle of embryo in each frame, the spatial position of the left half contour points of the embryo in each frame was calculated. Finally, the 3D point cloud of the embryo was obtained with high IoU [**Fig. S22B**].

**Methodologies for the surface area and volume calculation of reconstructed *C. elegans***

The surface area is calculated by summing the perimeters of the convex hulls for each 2D slice of the 3D point cloud. The steps are listed as follows:

- Step 1: Identify the points in the point cloud that share the same *y*-coordinate.
- Step 2: Project these points onto the *xz*-plane to form a 2D slice.
- Step 3: Compute the convex hull of these projected points to approximate the boundary of the shape in the *xz*-plane.
- Step 4: Calculate the perimeter of this convex hull.
- Step 5: Accumulate the surface area by summing the perimeters multiplied by the slice thickness.

$$Surface Area=\sum_{j} (\frac{Perimeter(j)}{fx\_scale})\times\frac{1}{fx\_scale}$$

where $Perimeter(j)$ is the perimeter of the convex hull for the slice at *y*-coordinate 𝑗, and $fx\_scale$ is the scaling factor.

The volume is calculated by integrating the area of each 2D slice across the *y*-axis. The steps involved are as follows:

- Step 1: Calculate the area of the convex hull formed by the points in the *xz*-plane.
- Step 2: Convert the area from pixel units to physical units by dividing by the square of the scaling factor.
- Step 3: Increment the volume by multiplying the area by the slice thickness.

The mathematical formula used is:

$$Volume=\sum_{j} (\frac{Area(j)}{{fx\_scale}^{2}})\times\frac{1}{fx\_scale}$$

where $Area(j)$ is the perimeter of the convex hull for the slice at y-coordinate 𝑗, and $fx\_scale$ is the scaling factor.

**Traditional 2D image processing methods for genetic interaction study.**

There exist irregularities in individual shapes of *C. elegans.* The estimated 3D models of each sample, which relies solely on the contours extracted from a randomly selected 2D image, is inaccurate [**Fig. S11**]. To prove the superiority of the developed system and the precisely 3D reconstructed models for 3D morphological phenotyping, we have included single 2D image-based 3D morphological phenotyping of the RNA binding protein mutants and N2 [**Fig. S30**], in which the 3D model reconstruction of each sample relies solely on single contour extracted from a randomly selected 2D image, in the revised supplementary file. The single contour in each 2D image was precisely extracted based on the machine learning technique. As shown in **Fig. S30A(i)**, no significant difference was observed in the surface area of embryos between wild type, single mutants, and double mutants. Thus, we cannot conclude that there existed a genetic interaction between *mbl-1* and *unc-75* in shaping the surface area of embryo. However, the genetic interaction between *mbl-1* and *unc-75* in shaping the surface area of embryo was identified by the precisely reconstructed 3D models as shown in Fig. 5. Also, as shown in **Fig. S30B(v)**, the genetic interaction between *mbl-1* and *unc-75* in influencing the ratio of length to maximum width of young adults could not be identified by the single 2D image-based analysis but was successfully recognized via the precisely reconstructed 3D models as shown in **Fig. 5**. Thus, precise 3D model reconstruction of each sample is quite of importance for the accurate 3D morphological phenotyping of *C. elegans*.

**
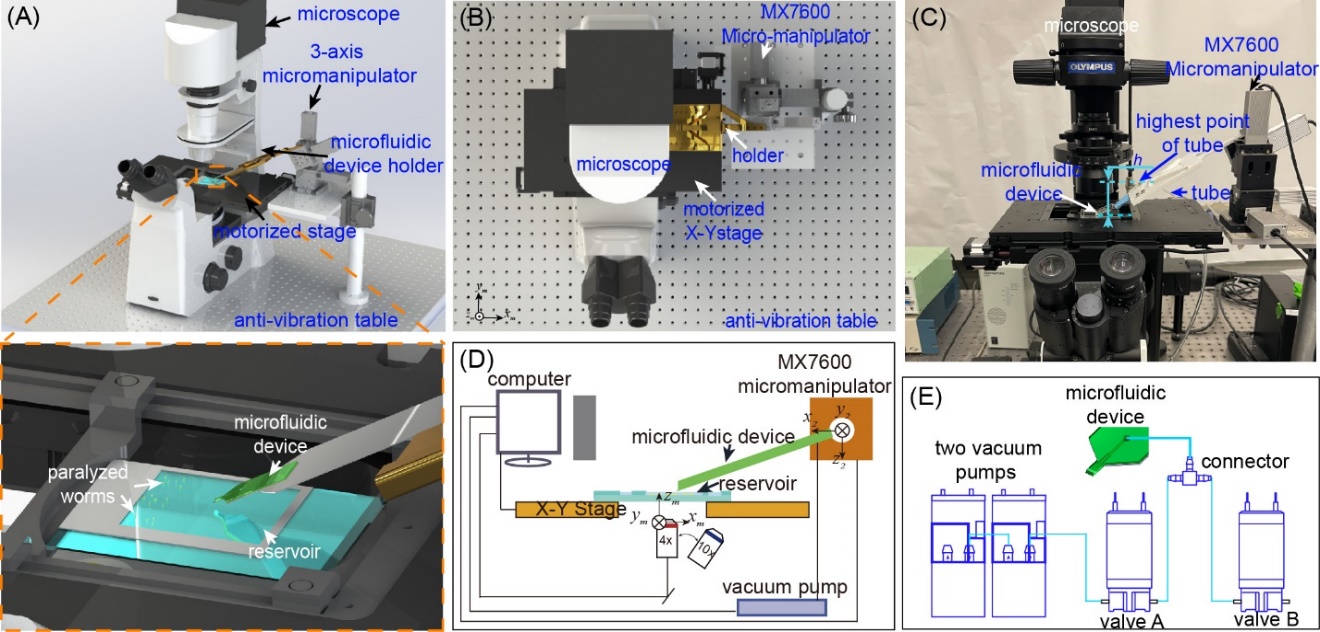
**

Fig. S1. Robotic system setup for the stable and uniform rotation of *C. elegans.* (A) Side view of the robotic system. (B) top view of the robotic system. (C) Photograph of the robotic system setup. (D) Schematic of the robotic system showing the main components. (E) Schematic of the pressure regulator system for the worm rotation.

**
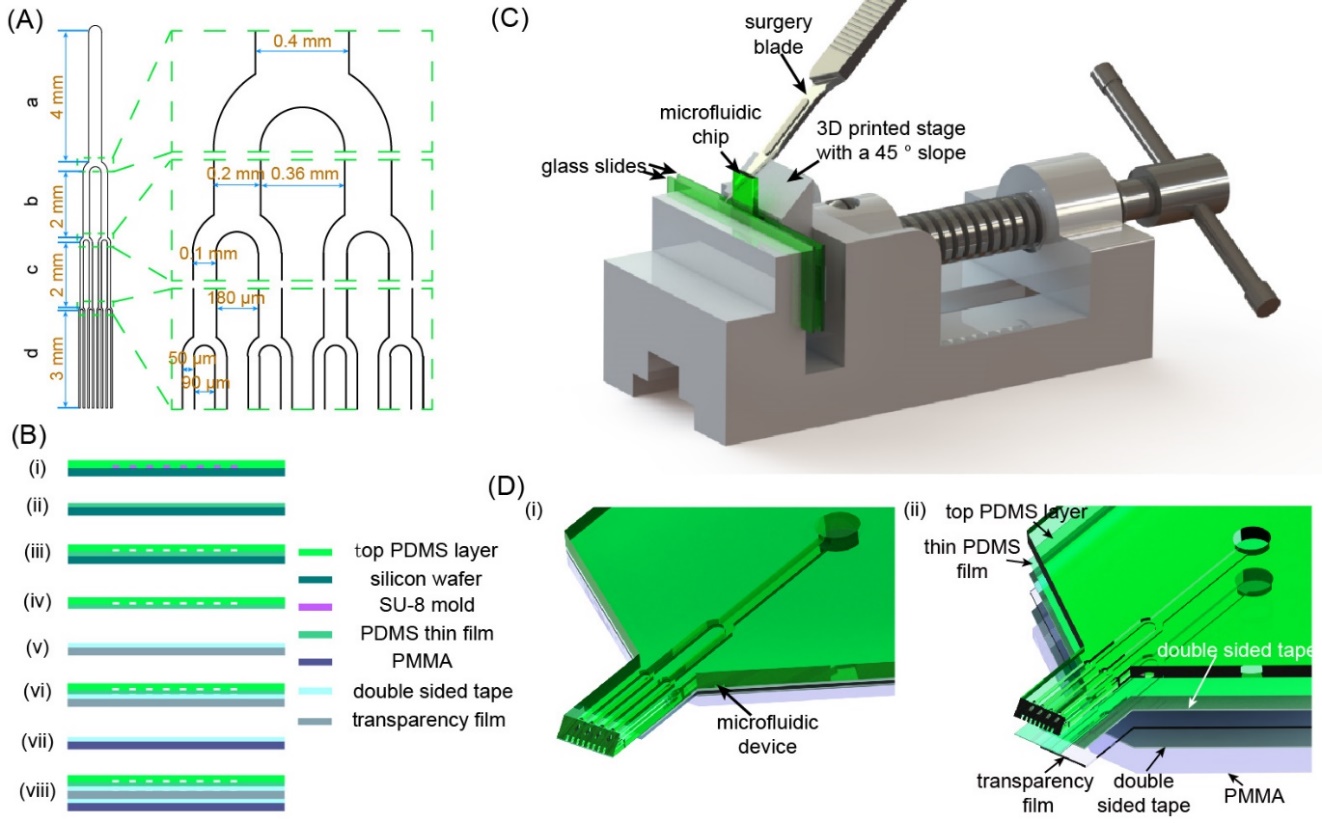
**

Fig. S2. Microfluidic device design and fabrication. (A) Detailed size of each microfluidic channel. (B) Workflow for the fabrication of the microfluidic device through the multilayer soft lithography. (C) Schematic of the experimental setup for obtaining the smooth slope of 45°at the side wall of the microfluidic device where the open ends of the microchannels are arranged. (D) Assembly of the microfluidic device and its exploded view.

**
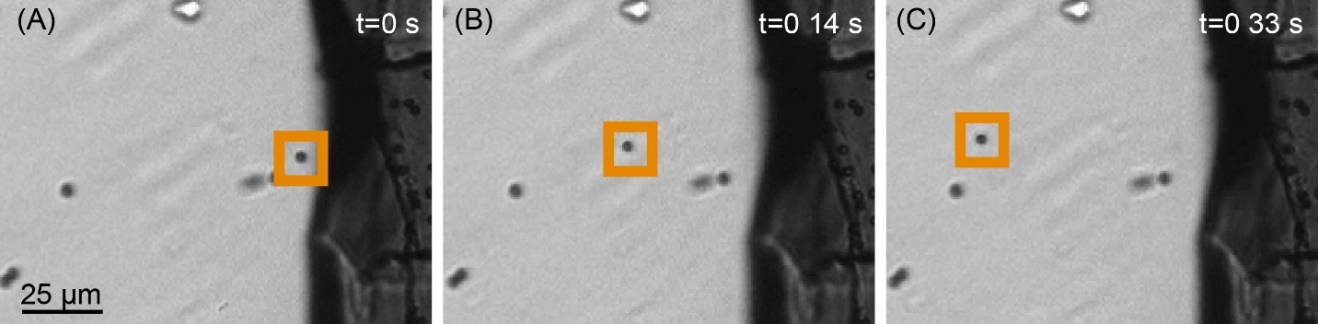
**

Fig. S3. Images showing the movement of a microparticle (3μm) at different time points when it just flows out of the microchannel with M9 medium under the action of gravity. (A) microparticle at the point of exiting the microchannel. (B) position of the microparticle at the time point of 0.14 s. (C) position of the microparticle at the time point of 0.33 s.

**
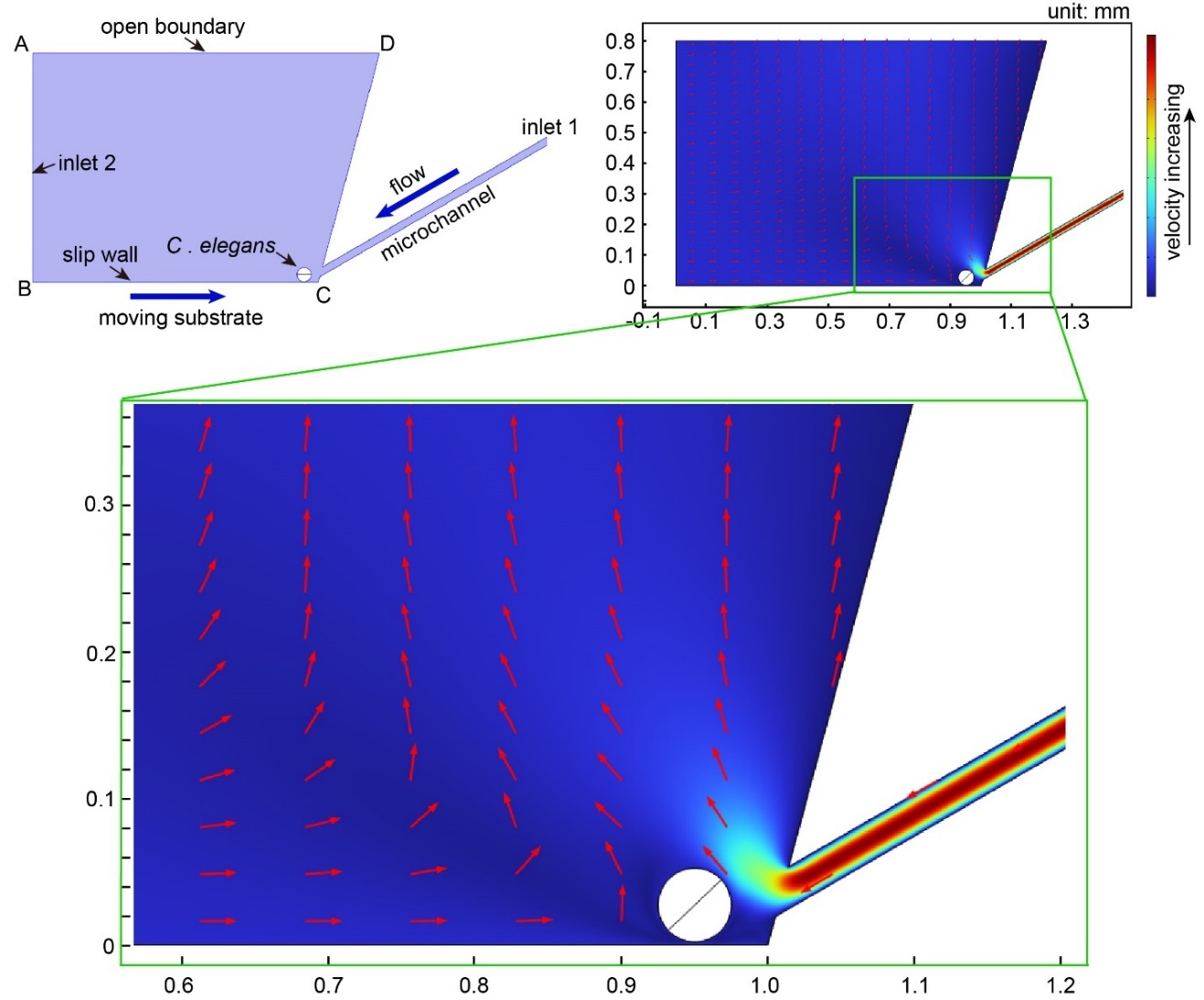
**

Fig. S4. Numerical simulation analysis of worm rotation.

**
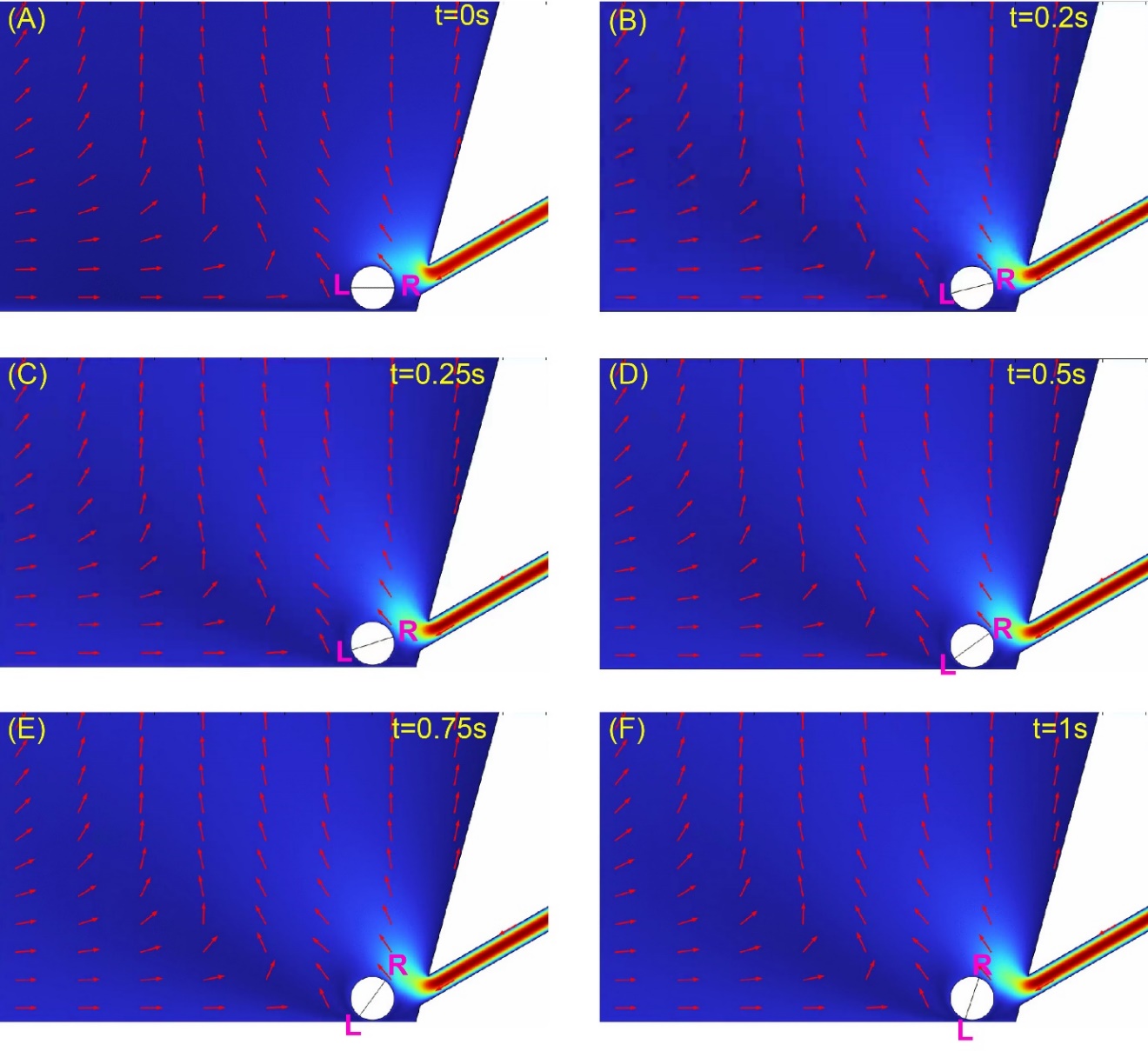
**

Fig. S5. Numerical simulation analysis showing the anti-clockwise rotation of C. elegans at different time points. (A)-(F) anti-clockwise rotation of *C. elegans.*


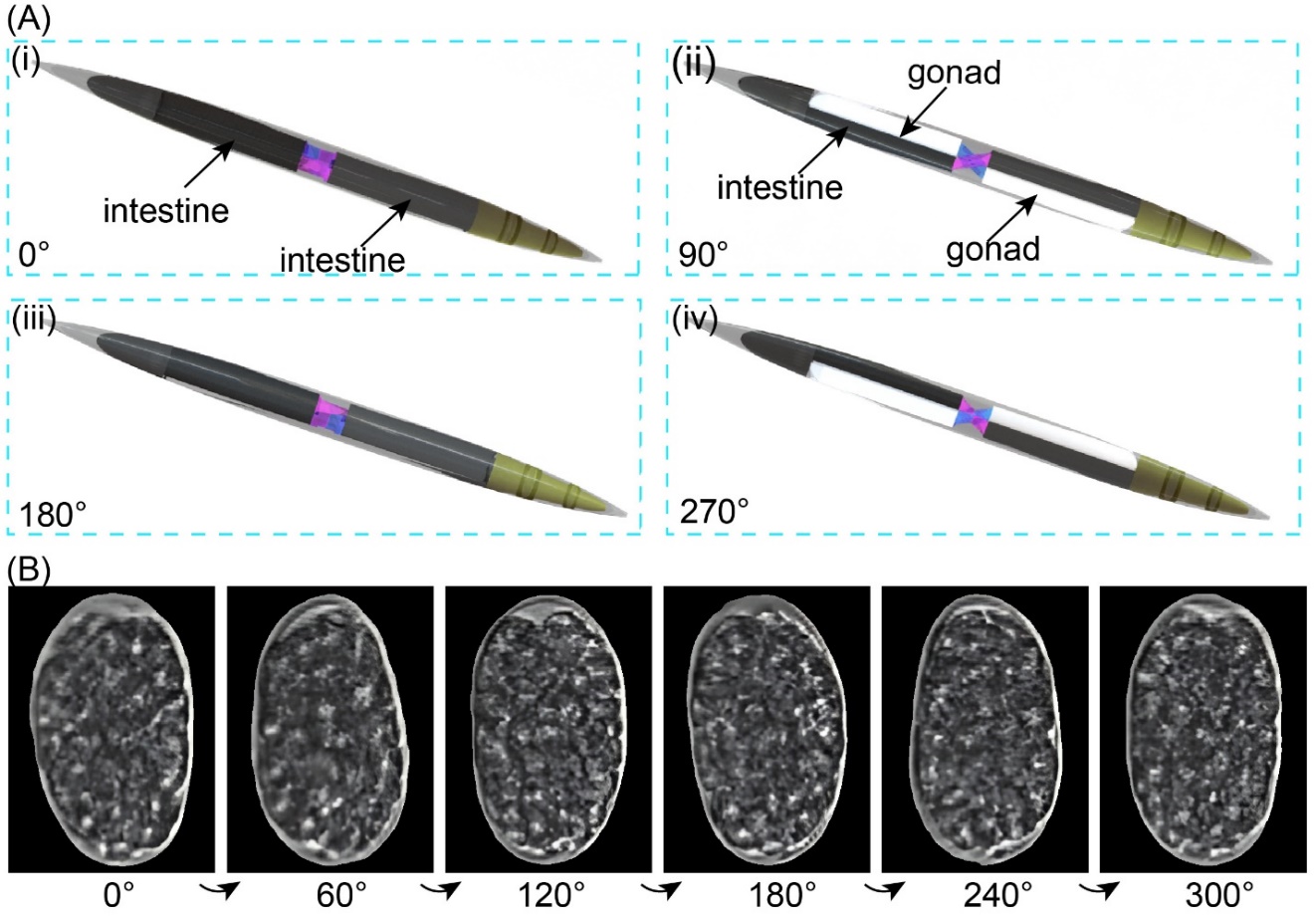


Fig. S6. Monitored rotation angle of worms by visually recognizing adult and embryo’s pattern. (A) Recognized pattern of the transparent gonad under a brightfield microscope. (B) Recognized morphological pattern of the embryo at different orientations


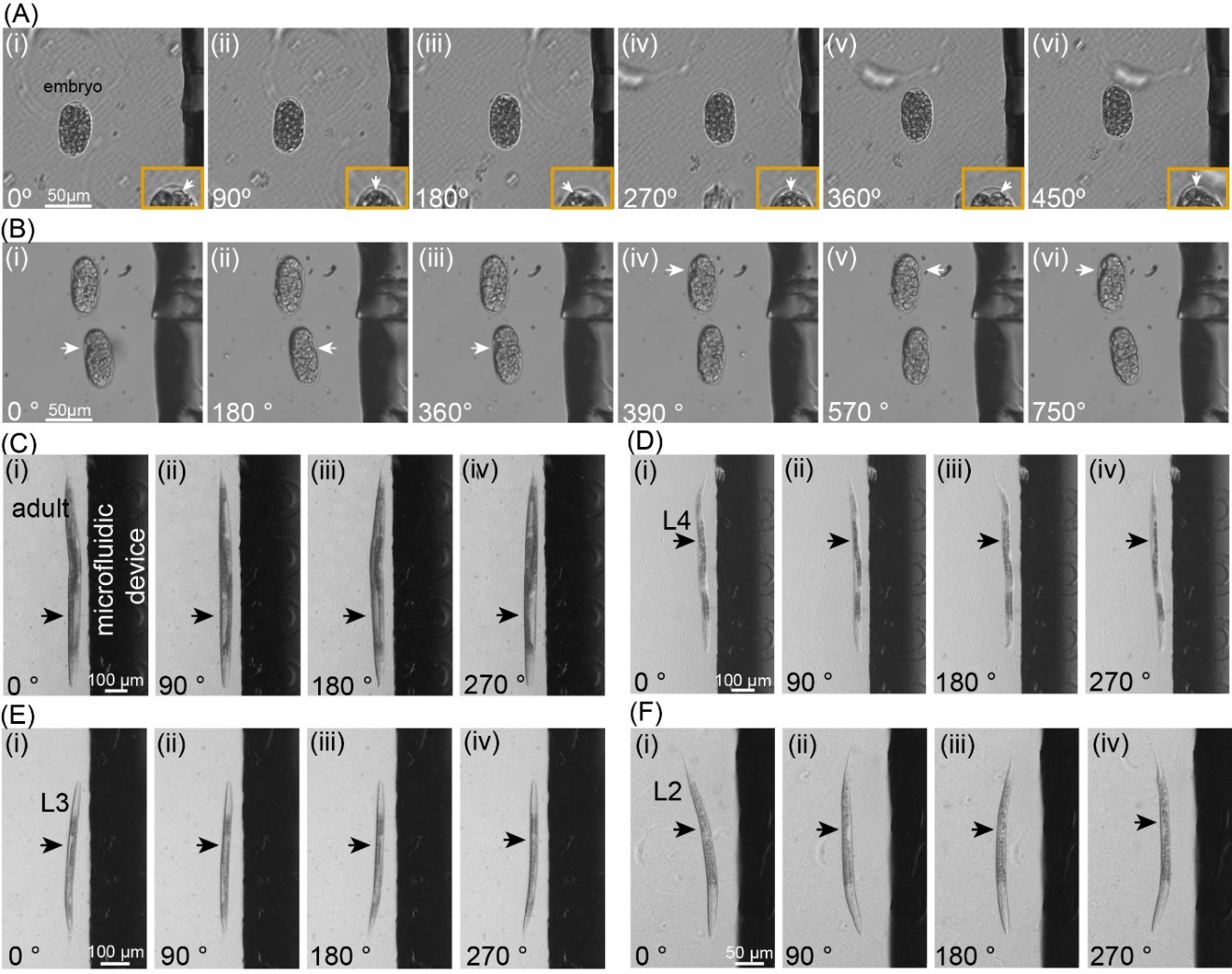


Fig. S7. Rotational manipulation of embryos, larvae, and adult worms. (A)-(B) Stable rotation of single/multiple embryos at different orientations. (C) Stable rotation of adult worms at different orientations. (D)-(F) Stable rotation of larvae ranging from L2 to L4 at different orientations.


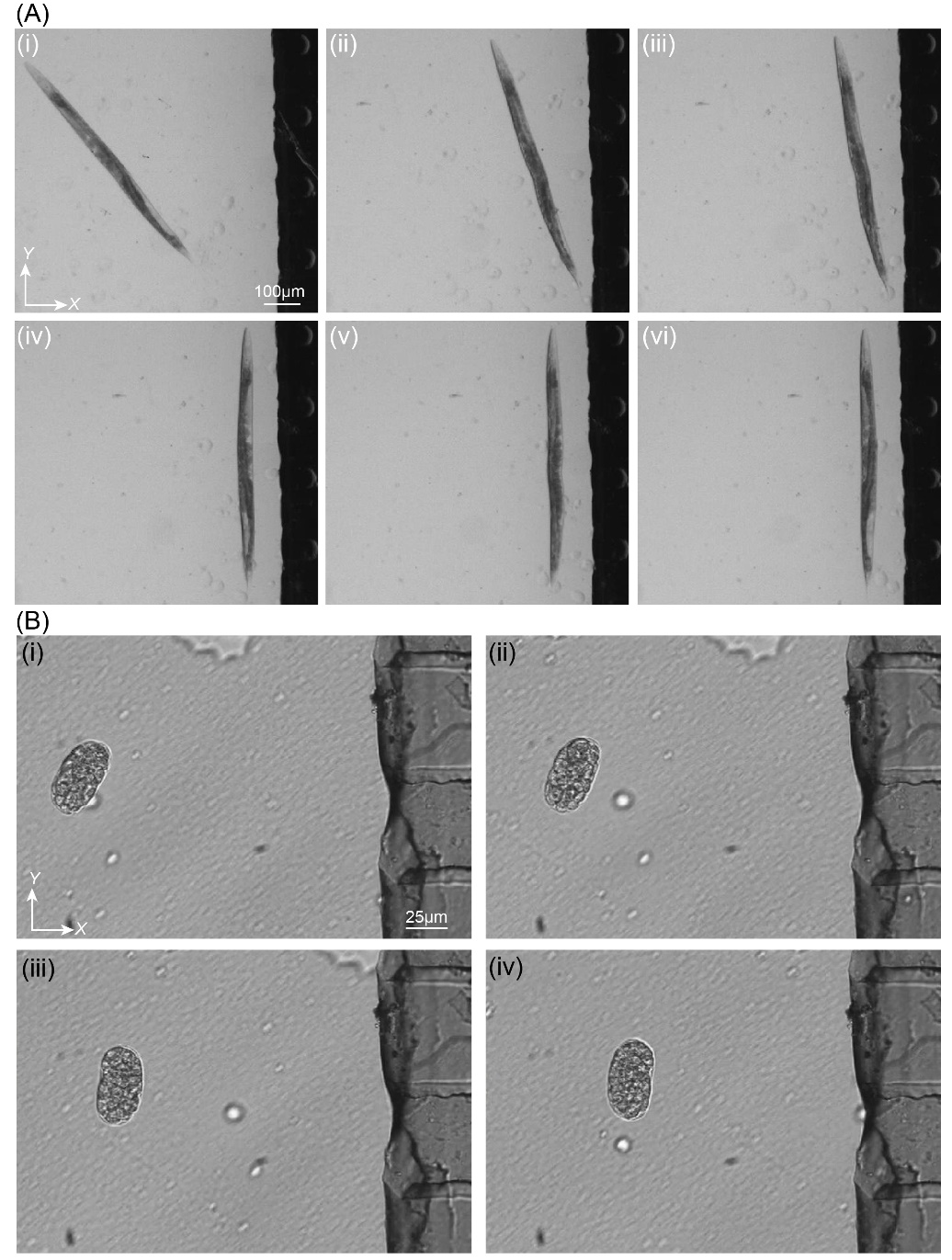


Fig. S8. Self-alignment of worms with the edge of the microfluidic device via rotation around the Z-axis. (A) Self-alignment of an adult with the edge of the microfluidic device. (B) Self-alignment of an embryo with the edge of the microfluidic device.

**
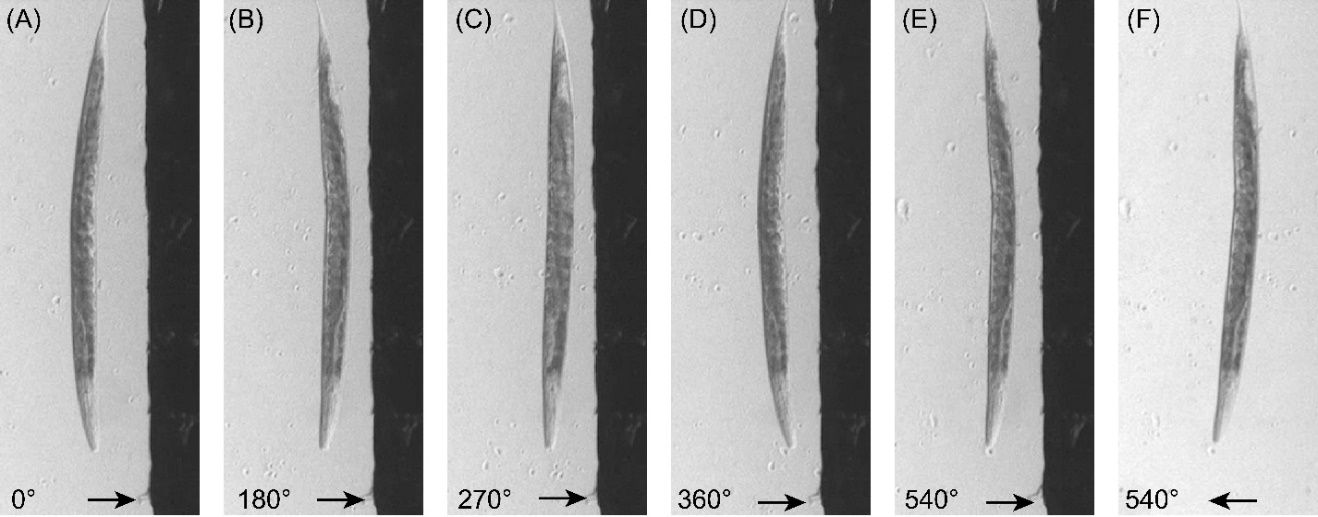
**

Fig. S9. Stable stoppage of worm rotation once it is rotated to the desired orientation. (A)-(E) Continuous rotation of worm at different orientations. (F) Stable stoppage of worm rotation by moving the worm leftwards at a relatively large speed.


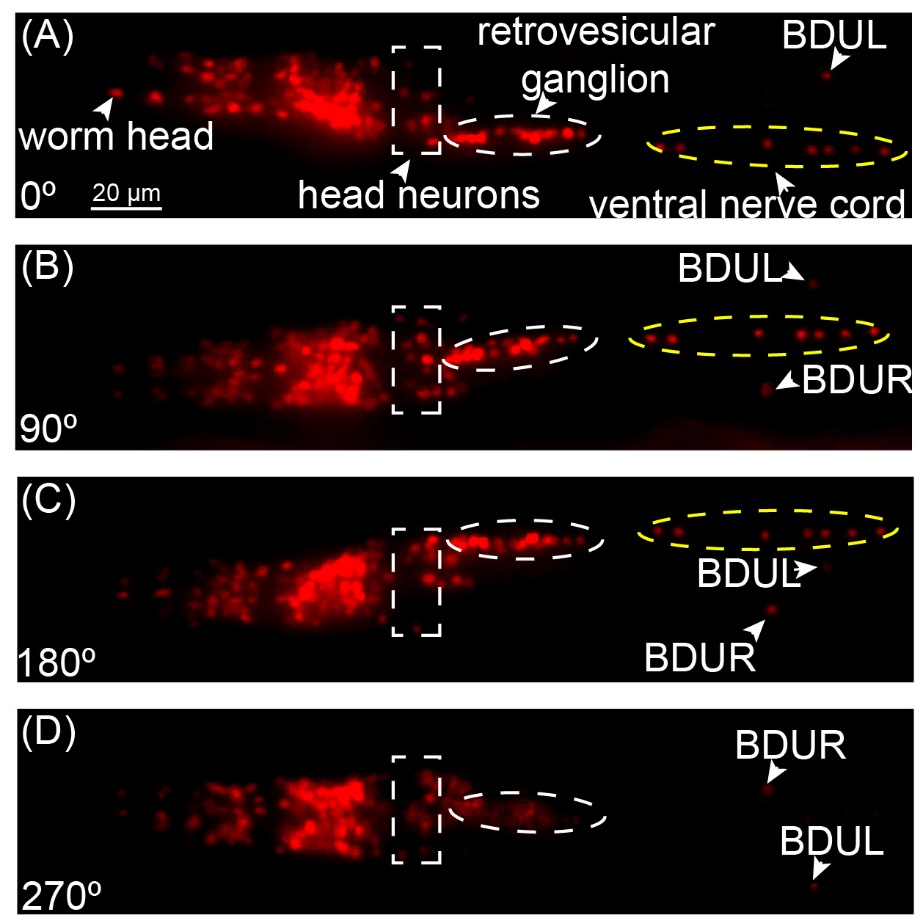


Fig. S10. Fluorescent Imaging of all head neurons in the anterior part of *C. elegans* (JAC769) from different orientations. (A) Fluorescent Imaging at the orientation of 0°. (B) Fluorescent Imaging at the orientation of 90°. (C) Fluorescent Imaging at the orientation of 180°. (D) Fluorescent Imaging at the orientation of 270°.

**
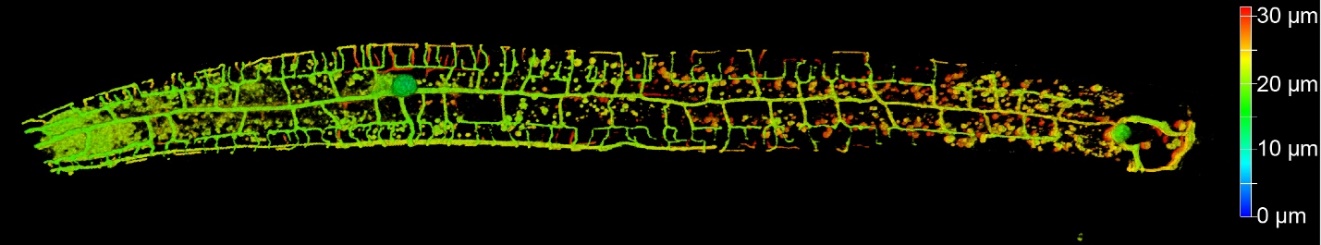
**

Fig. S11. Incomplete 3D model reconstructed from the confocal imaging of PVD neurons of the worm (NC1686) when using the standard laser scanning confocal microscopy.

**
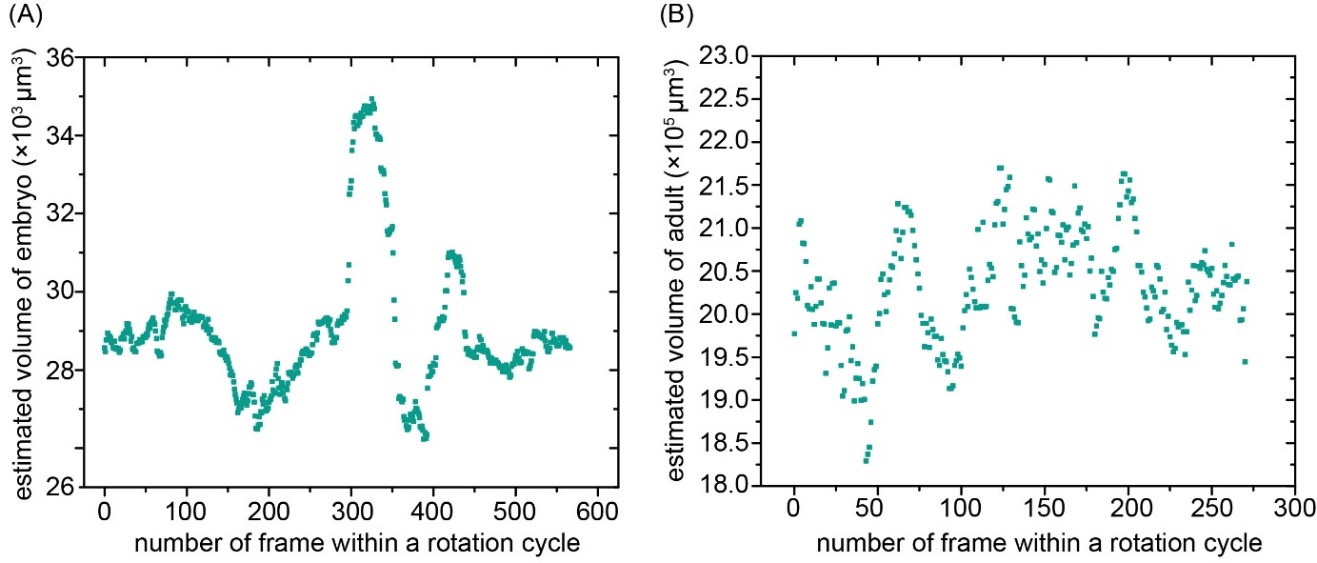
**

Fig. S12. Estimated volume of *C. elegans* based on single 2D images obtained at different orientations within a rotation cycle. (A) Estimated volume of the same embryo. (B) Estimated volume of the same adult.


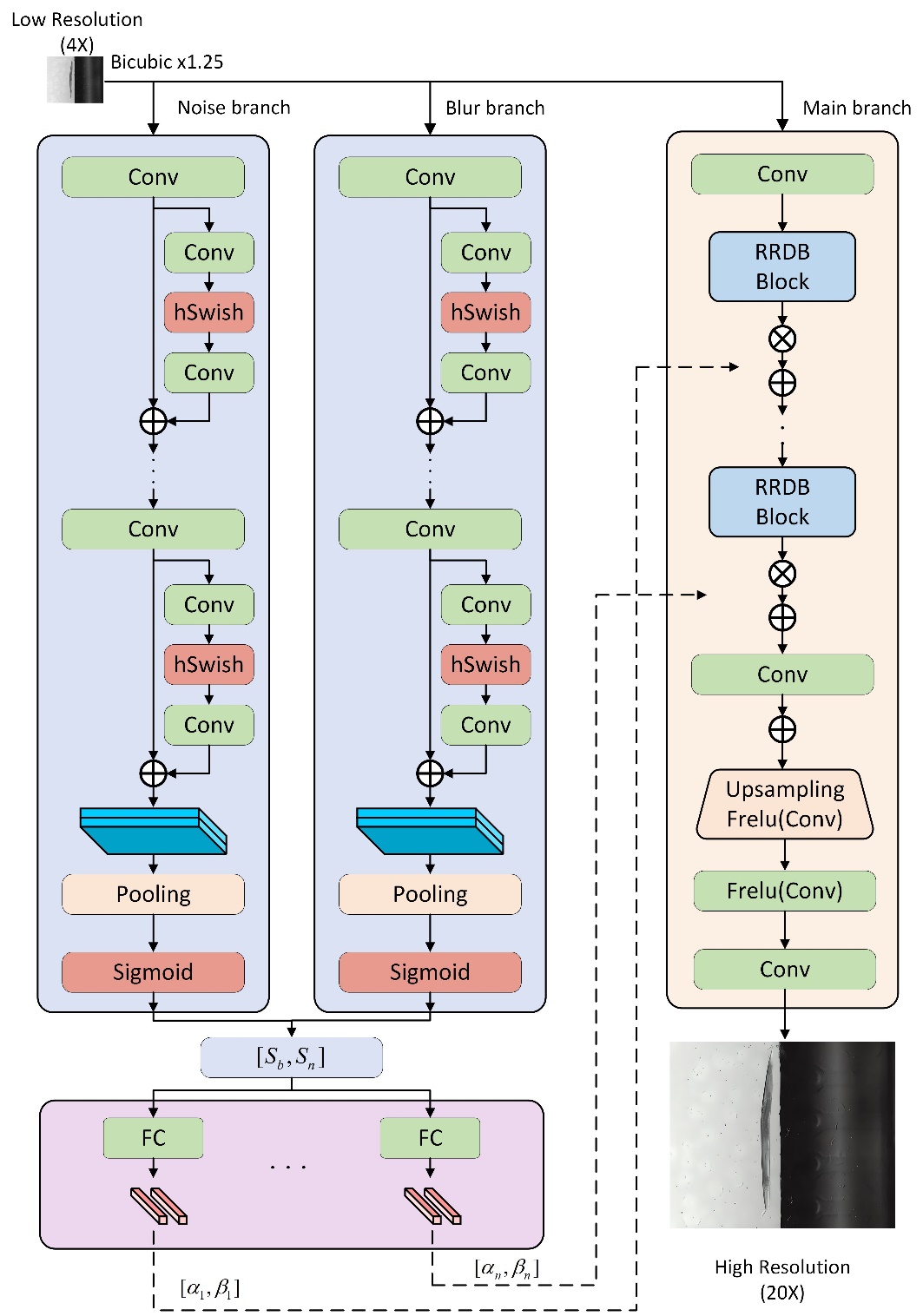


Fig. S13. The architecture of super resolution neural network.


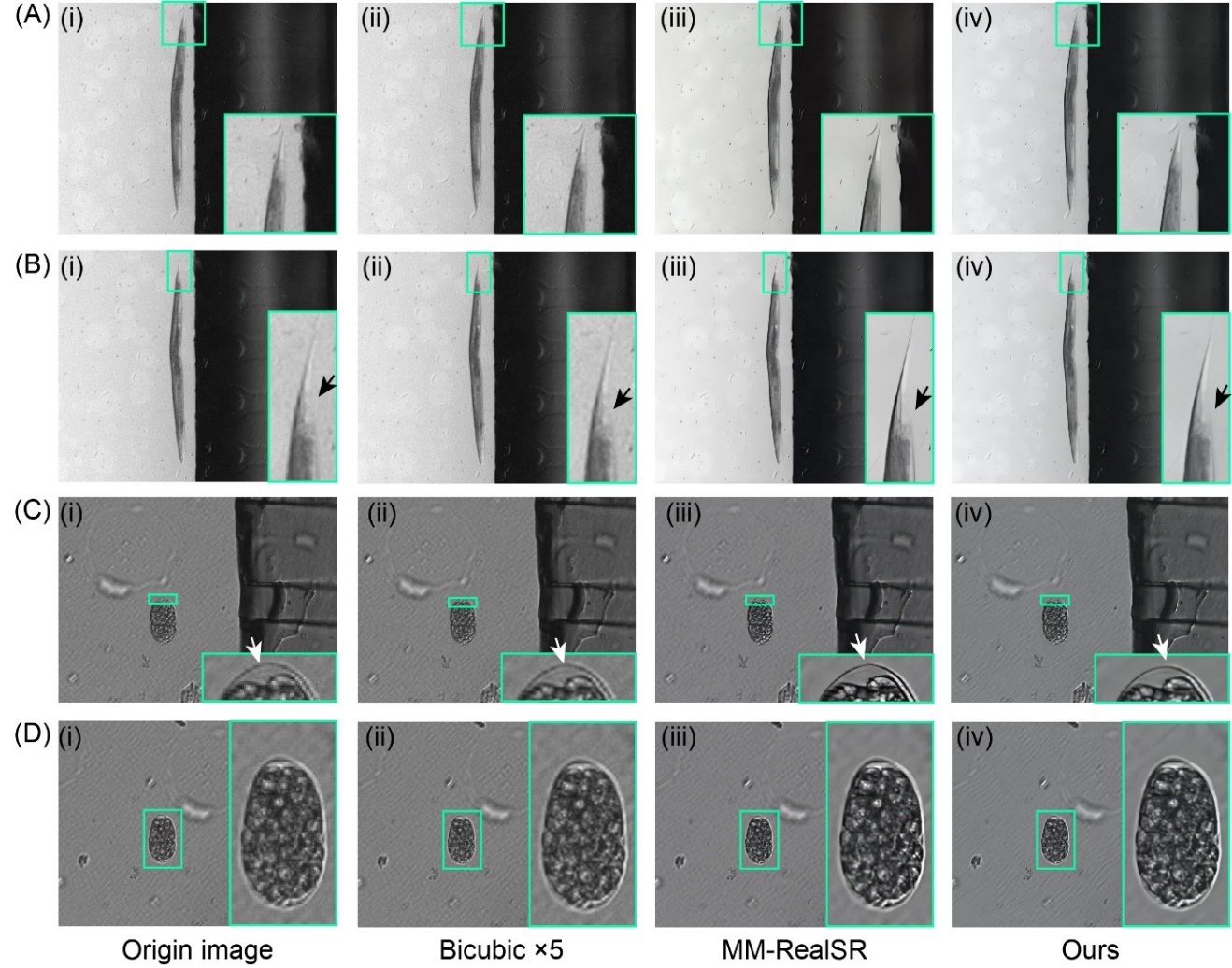


Fig. S14. Super resolution of images using our proposed method and other methods. (A)-(B) Super resolution and denoising of adult images by using different methods. (C)-(D) Super resolution and denoising of embryo images by using different methods.


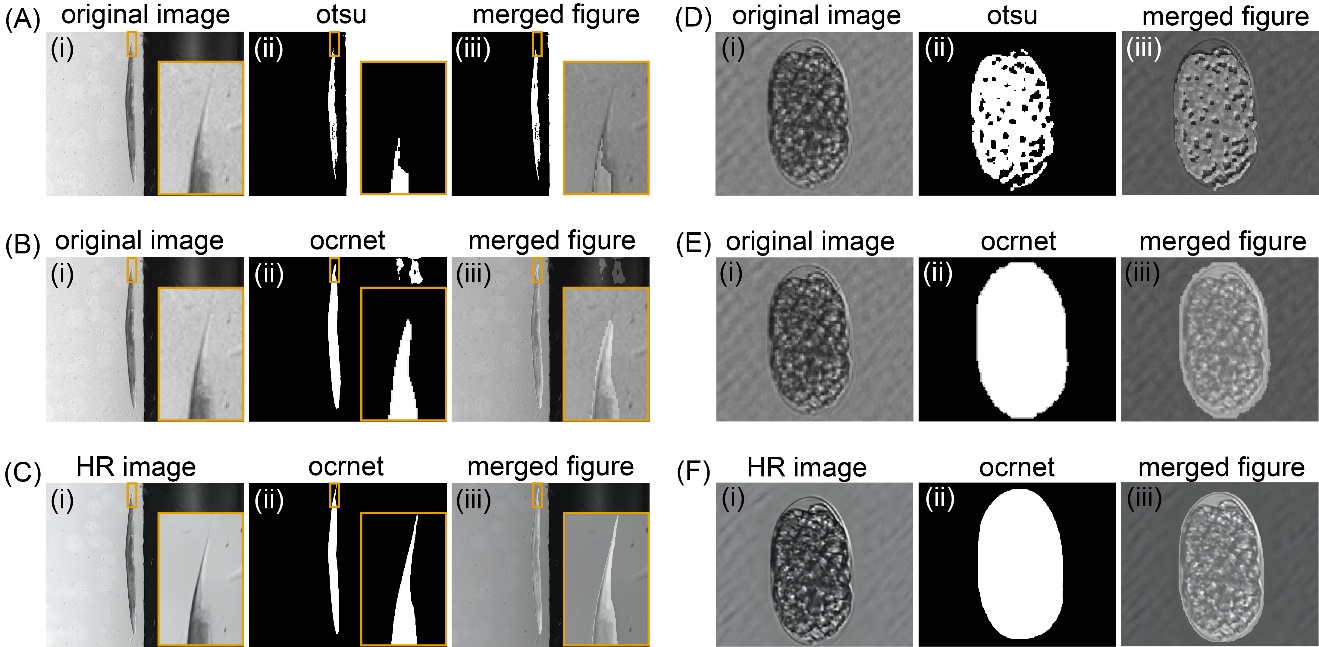


Fig. S15. Segmentation of worms using different methods. (A) Adult segmentation in original microscopy image using OTSU method. (B) Adult segmentation in original microscopy image using semantic segmentation approach. (C) Adult segmentation in high resolution image using semantic segmentation approach. (D) Embryo segmentation in original microscopy image using OTSU method. (E) Embryo segmentation in original microscopy image using semantic segmentation approach. (F) Embryo segmentation in high resolution image using semantic segmentation approach.


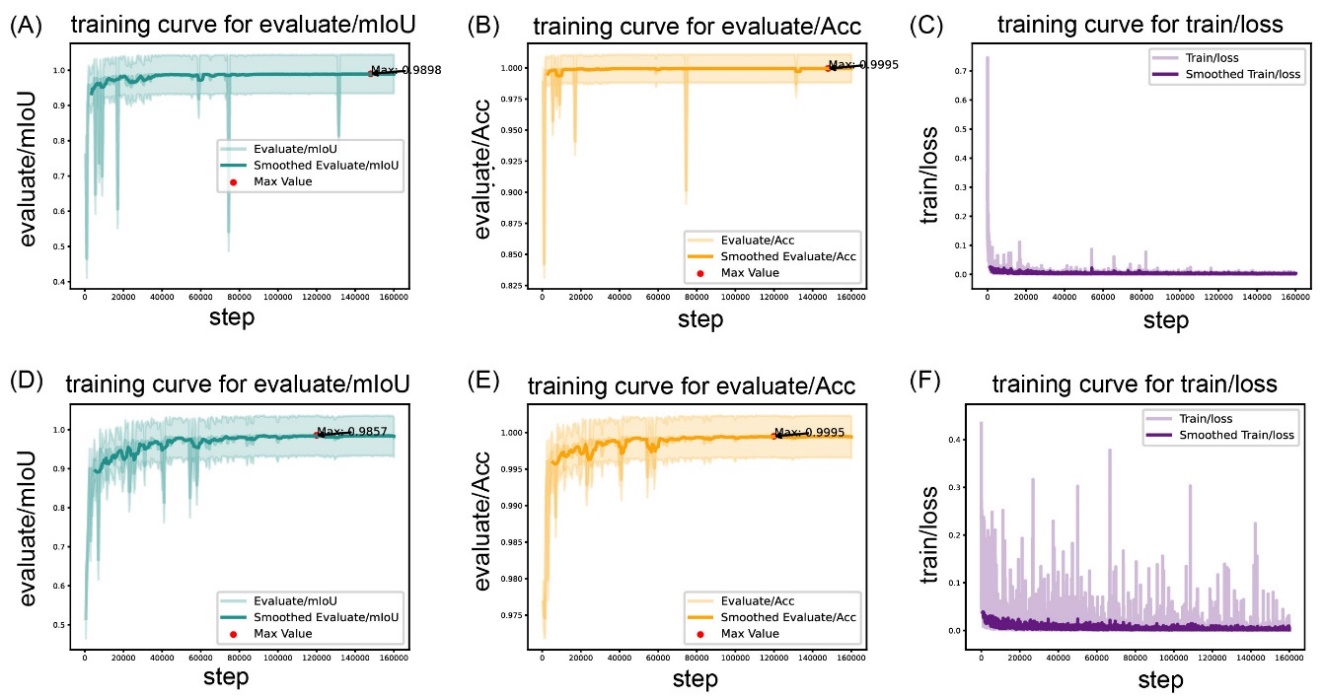


Fig. S16. Training process of segmentation. (A)-(C) Training curve of adult segmentation. (D)-(F) Training curve of embryo segmentation.


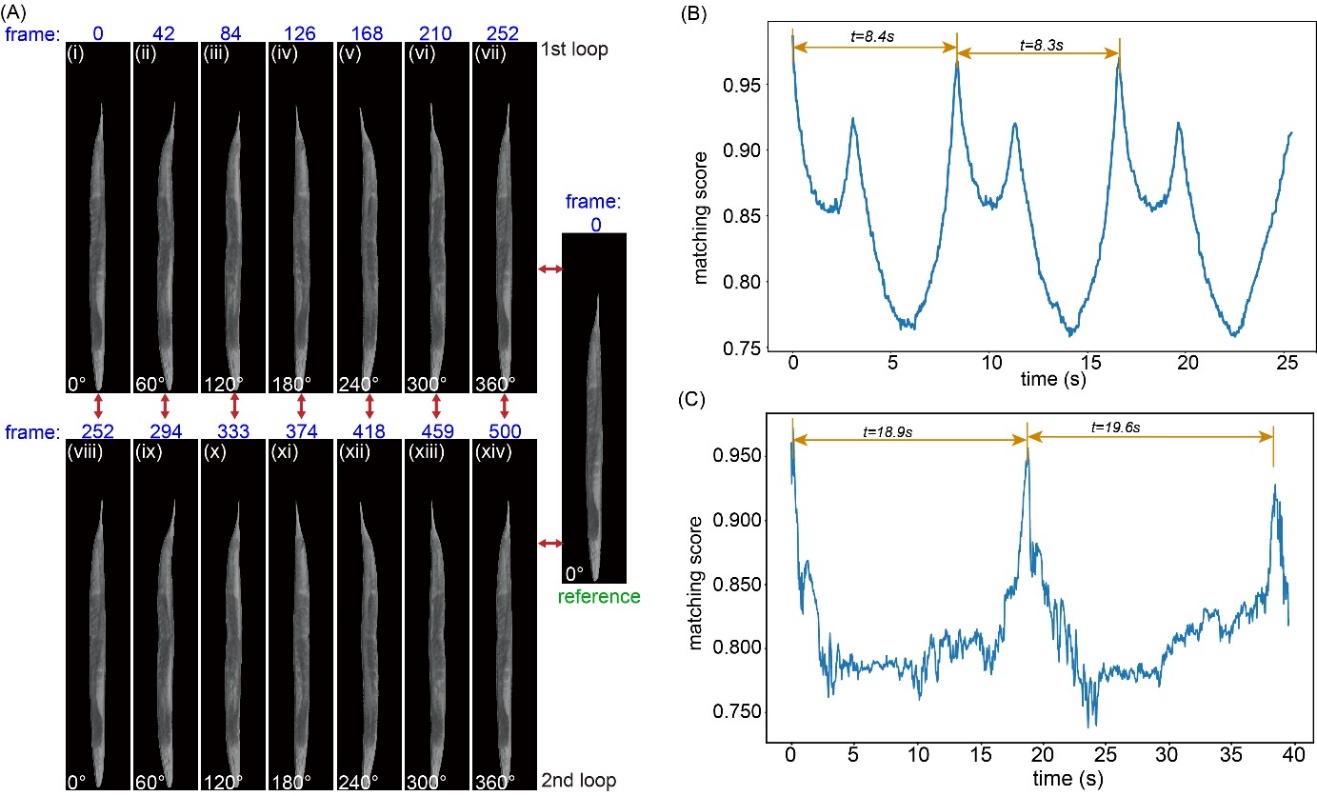


Fig. S17. Accurate measurement of *C. elegans* rotation cycle. (A) Images of rotated young adult worms at different orientations within two rotation cycles. (B) Measurement of young adult rotation cycle based on the template matching algorithm. (C) Measurement of embryo rotation cycle based on the template matching algorithm.


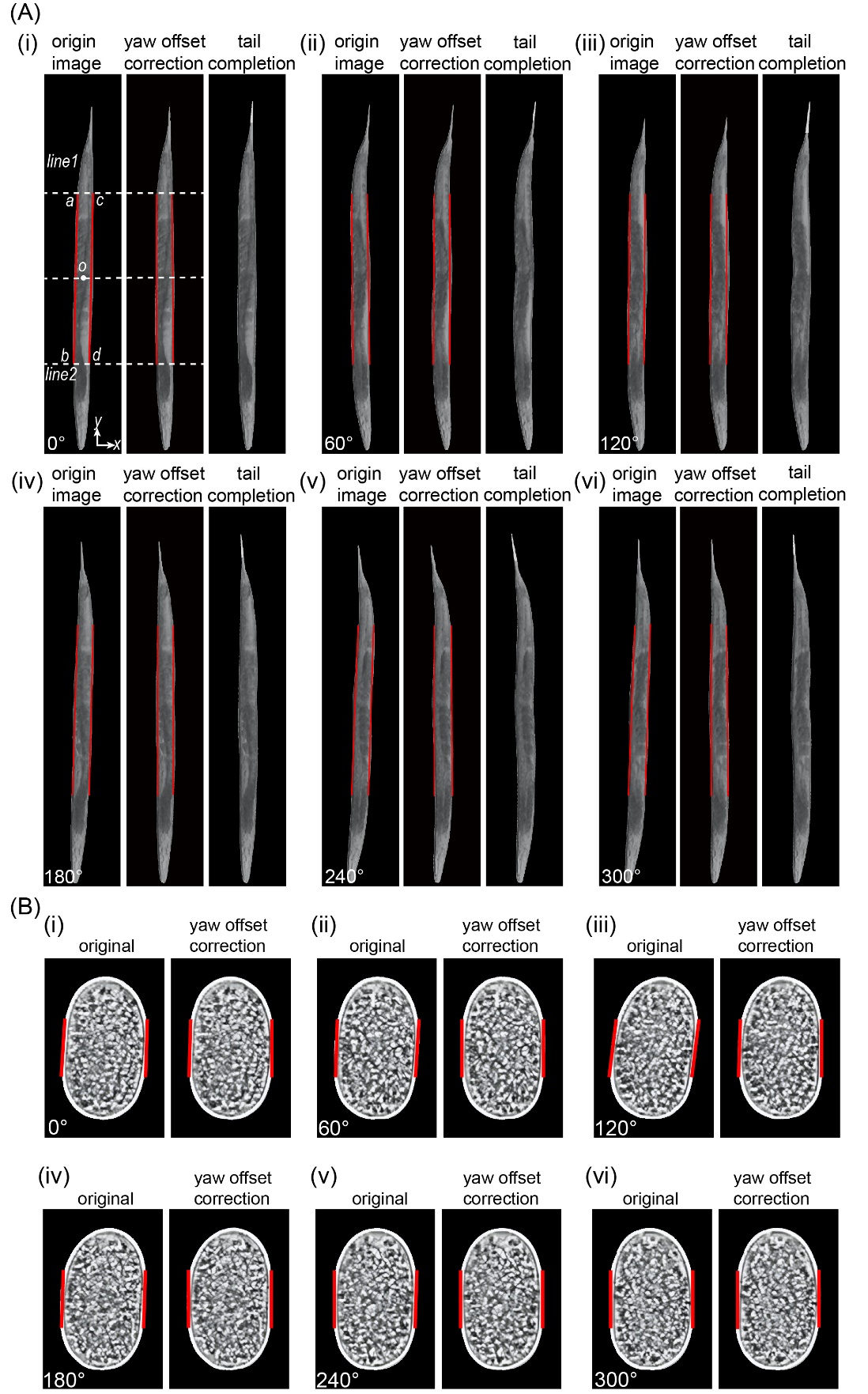


Fig. S18. Alignment of *C. elegans* for precise 3D reconstruction. (A) Alignment of adult worm at different orientations. (B) Alignment of embryo at different orientations.


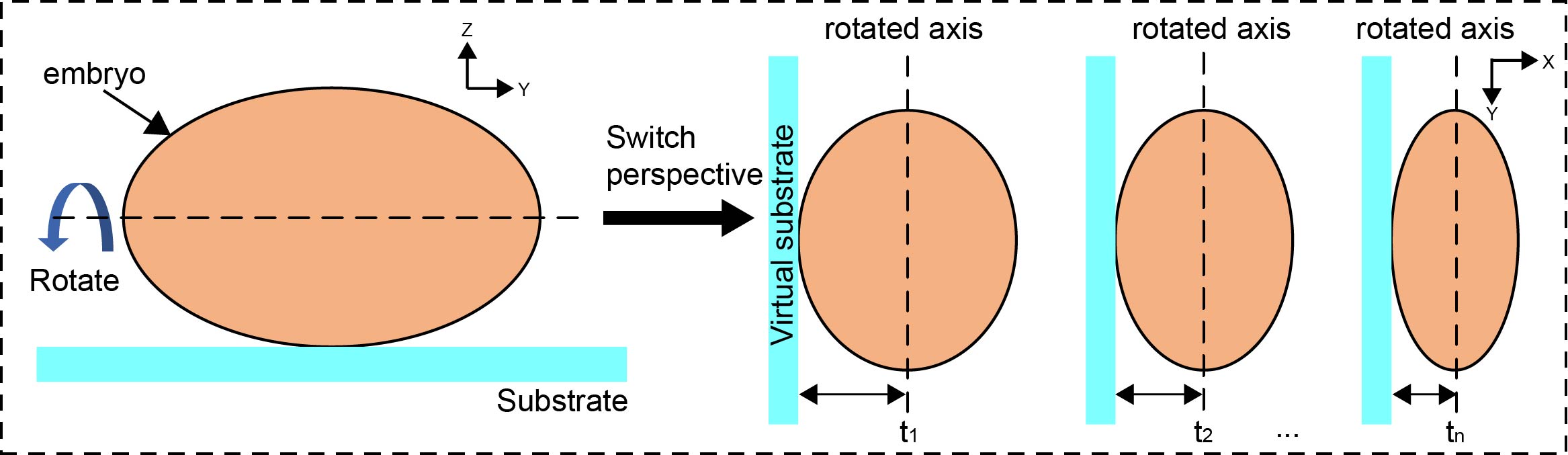


Fig. S19. Embryo position alignment and rotation axis calculation.

**
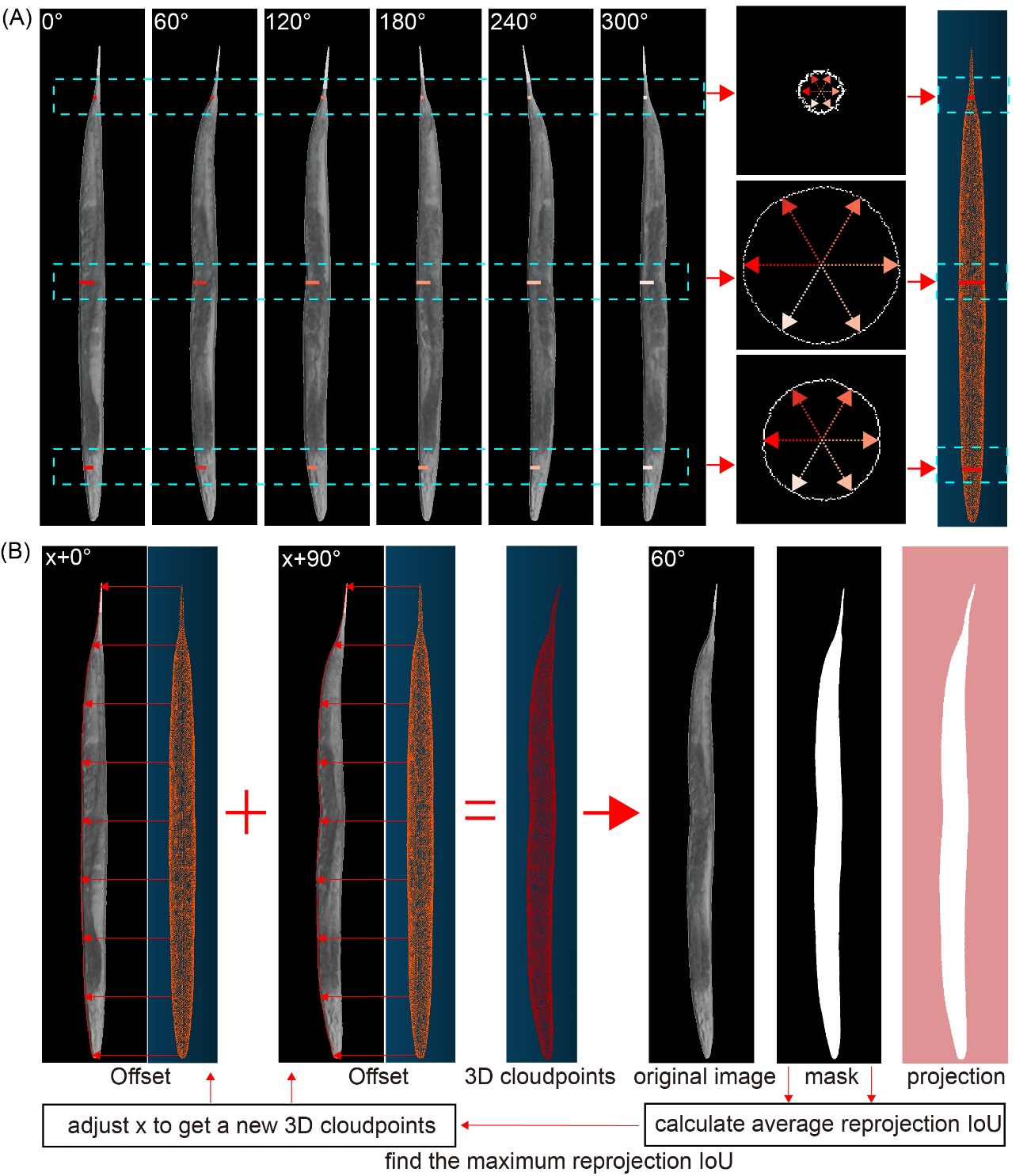
**

Fig. S20. Methodologies for 3D reconstruction of adult worms. (A) Generation of the preliminary 3D point cloud of adult worms. (B) Optimization of the 3D point cloud of adult worms.


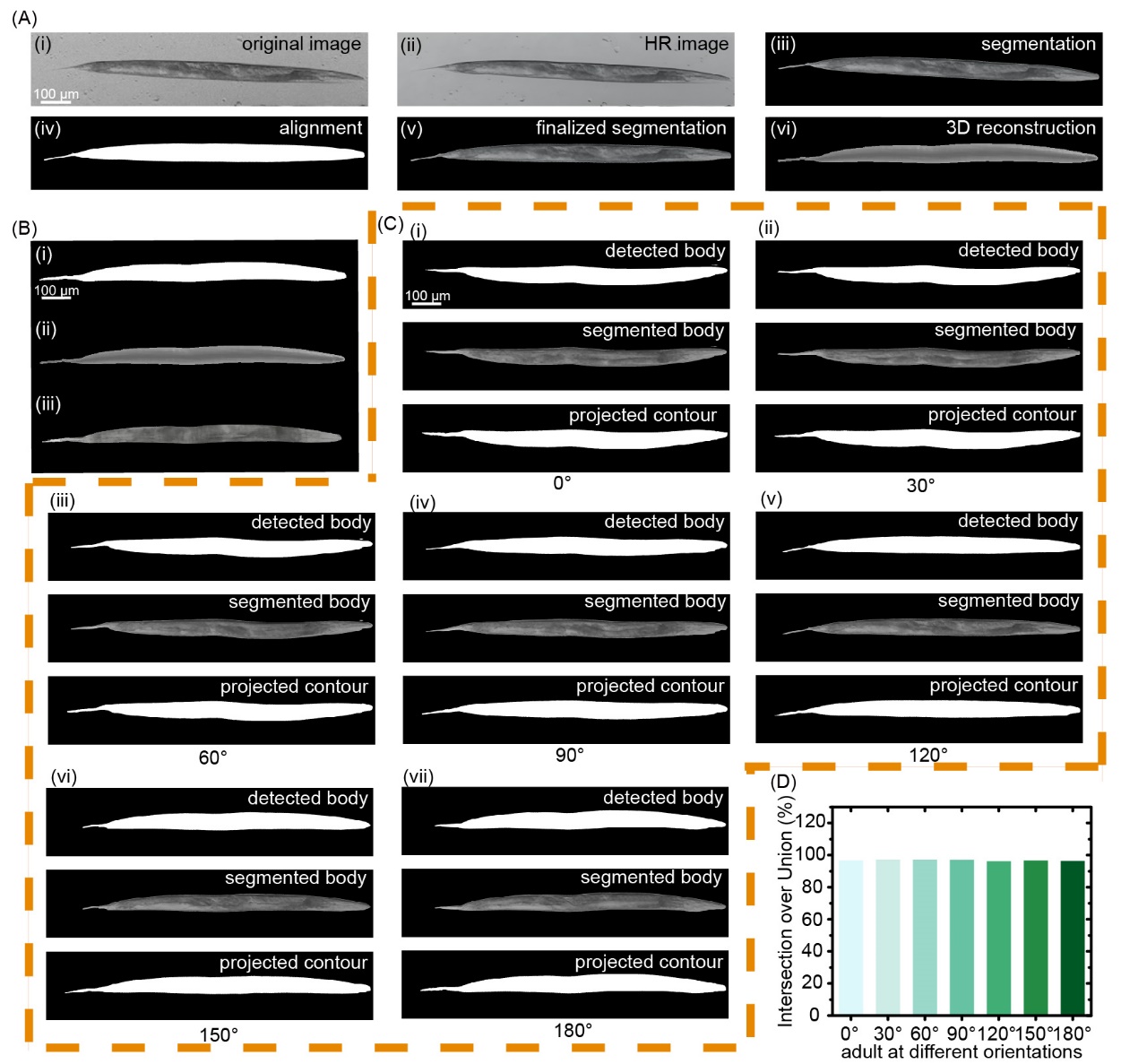


Fig. S21. 3D reconstructed models of adult worm from large collection of 2D images. (A) Pre-processed steps for the 3D reconstruction: (i) acquisition of original microscopy images, (ii) super-resolution image, (iii) worm detection, (iv) segmentation, (v) in-plane alignment of detected worm body, (vi) segmentation of aligned worm body. (B) 3D reconstruction of an adult worm: (i) point cloud-based 3D model, (ii) 3D model with reconstructed surface, (iii) 3D model with texture mapping. (C) Comparison between the projected images of 3D model and the captured microscope image at same orientations. (D) IoU validation for the reconstructed 3D model of adult worm at different orientations.

**
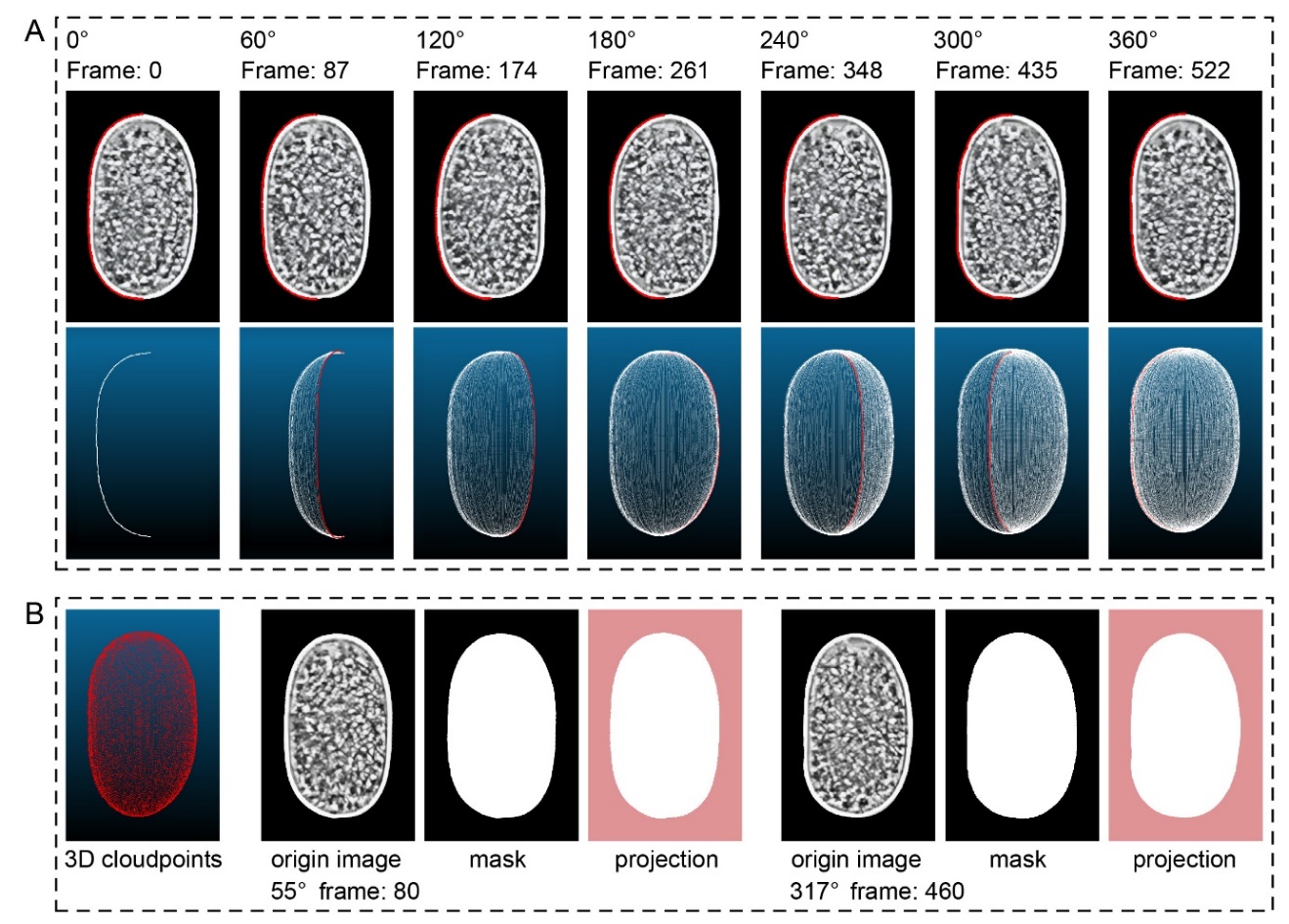
**

Fig. S22. Methodologies for 3D reconstruction of embryos. (A) Generation of the 3D point cloud of embryos. (B) Comparison between the original images and the projected images of 3D reconstructed model.


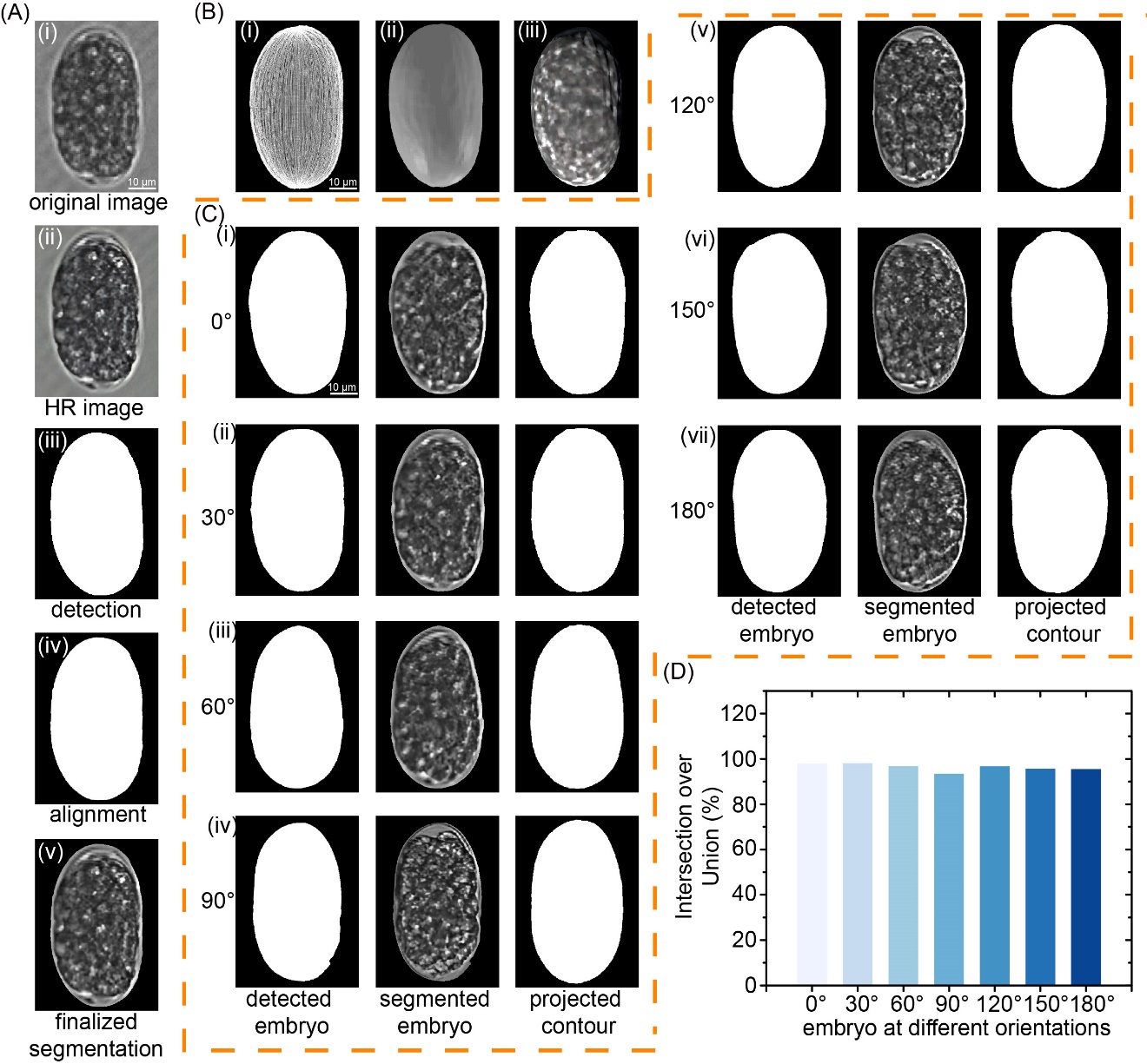


Fig. S23. 3D reconstructed models of embryos from large collection of 2D images. (A) Pre-processing steps for the 3D reconstruction (i) acquisition of original microscopy image, (ii) image super-resolution, (iii) embryo detection, (iv) in-plane alignment of detected embryo, (v) segmentation of aligned embryo. (B) 3D reconstruction of embryo: (i) point cloud-based 3D model, (ii) 3D model with reconstructed surface, (iii) 3D model with texture mapping. (C) Comparison between the projected images and captured microscope image at the orientation of 0 °. (D) IoU validation for the reconstructed 3D model of embryo at different orientations.


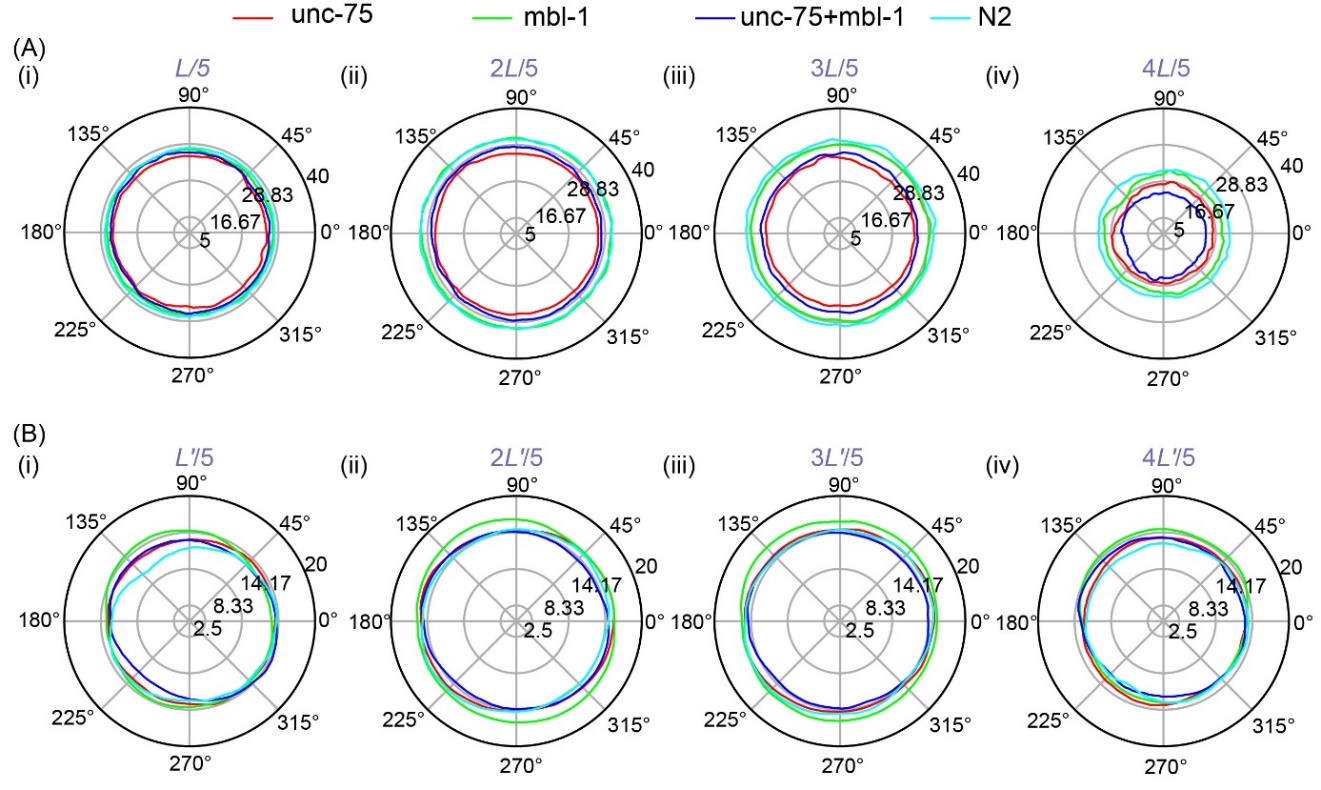


Fig. S24. Outline analysis of the transverse section of young adults and embryos along the worm body from different strains. (A) Transverse section outlines of young adults at different positions in which the ratio of the distance to the whole length of young adults was same (*L* represents the length of young adult worm): (i) transverse section outlines at the position of *L*/5 starting from the adult head. (ii) transverse section outlines at the position of 2*L*/5 starting from the adult head. (iii) transverse section outlines at the position of 3*L*/5 starting from the adult head. (iii) transverse section outlines at the position of 3*L*/5 starting from the adult head. (B) Transverse section outlines of embryos at different positions (*L'* represents the length of young adult worm): (i) transverse section outlines at the position of *L'*/5 starting from the topmost point of the aligned embryo. (ii) transverse section outlines at the position of 2*L'*/5 starting from the topmost point of the aligned embryo. (iii) transverse section outlines at the position of 3*L'*/5 starting from the topmost point of the aligned embryo. (iii) transverse section outlines at the position of 4*L'*/5 starting from the topmost point of the aligned embryo.


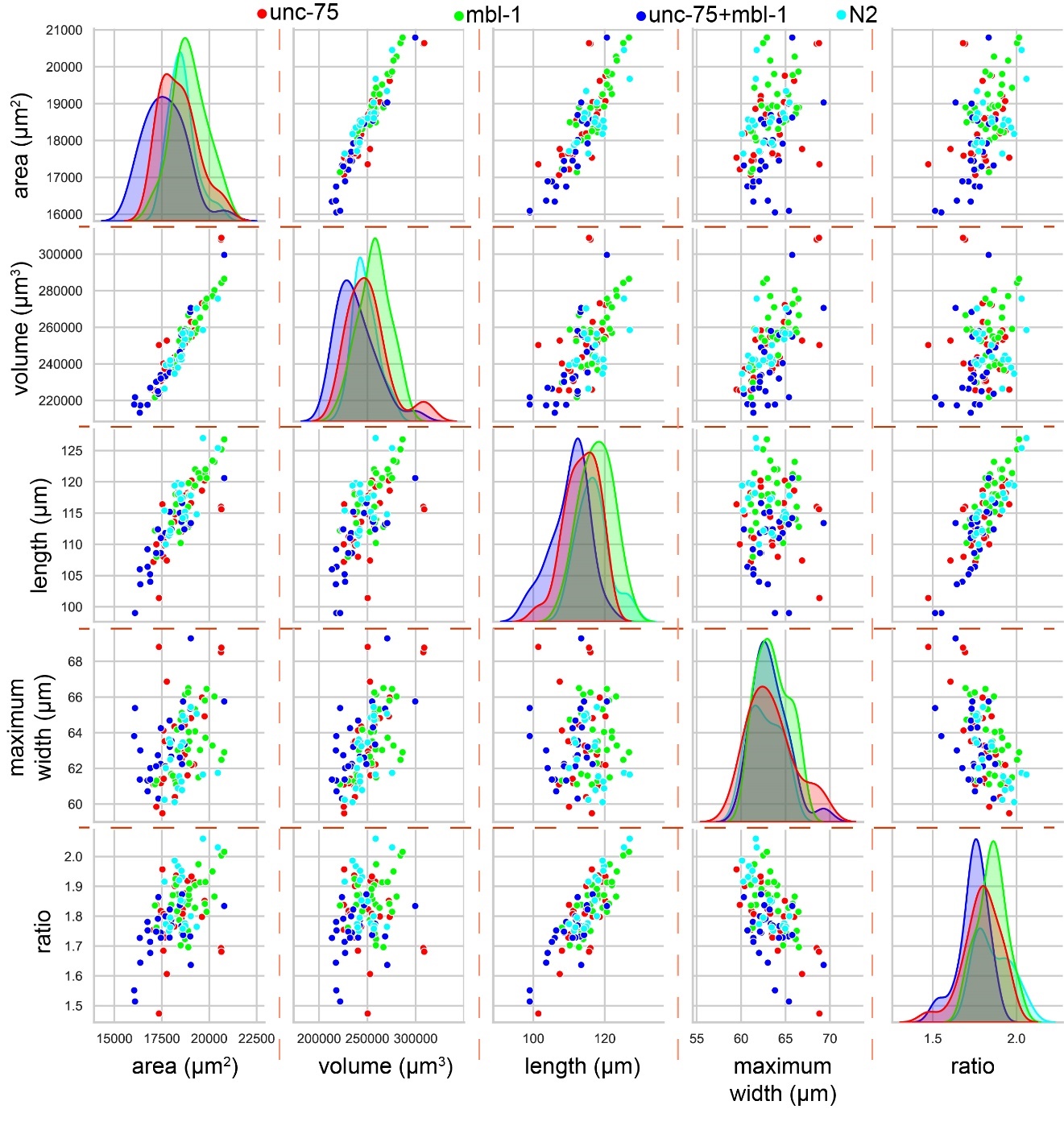


Fig. S25. Correlation relationship between different morphological metrics of embryos from different strains.


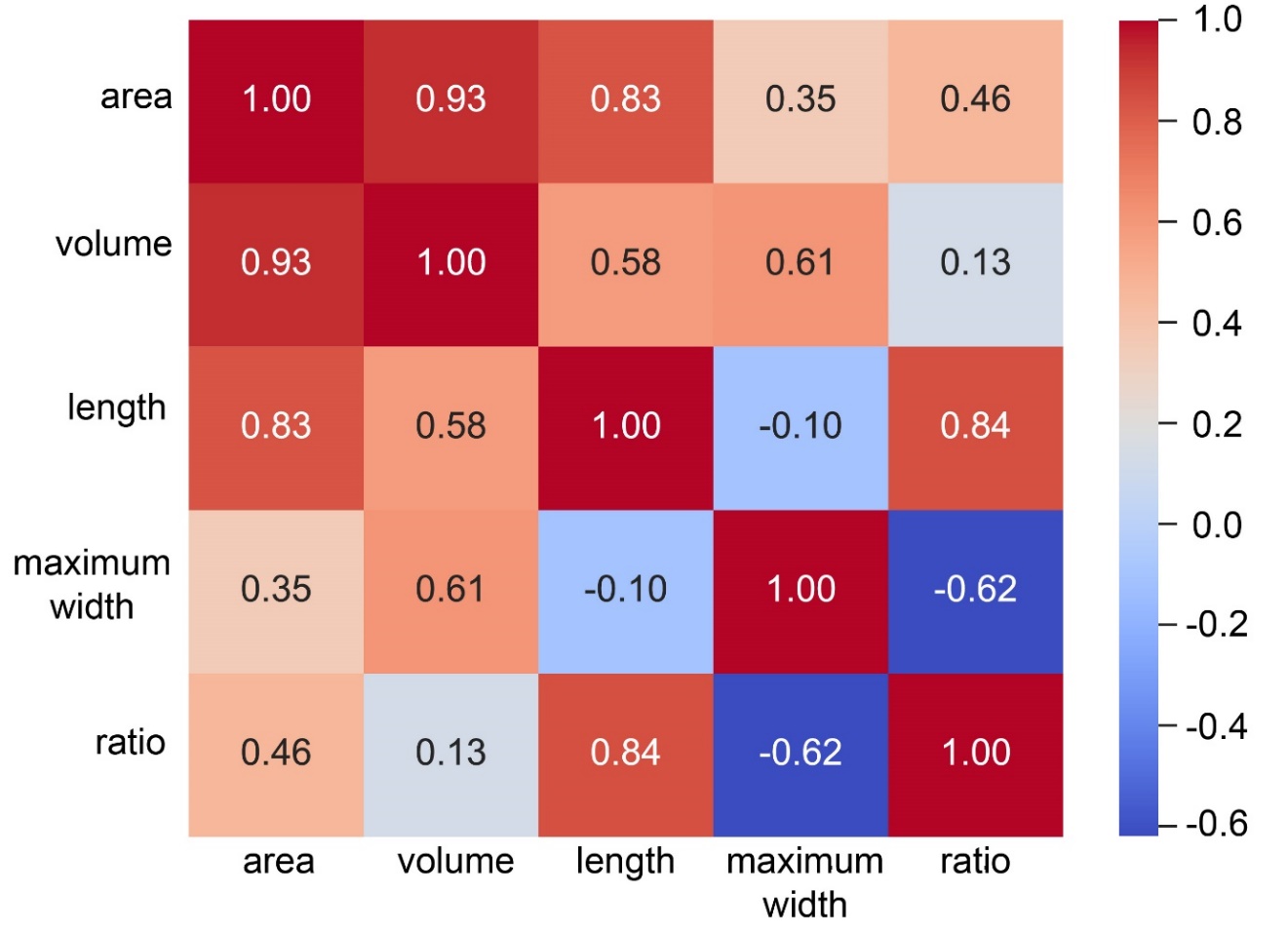


Fig. S26. Correlation matrix between different morphological metrics of embryos from different strains.


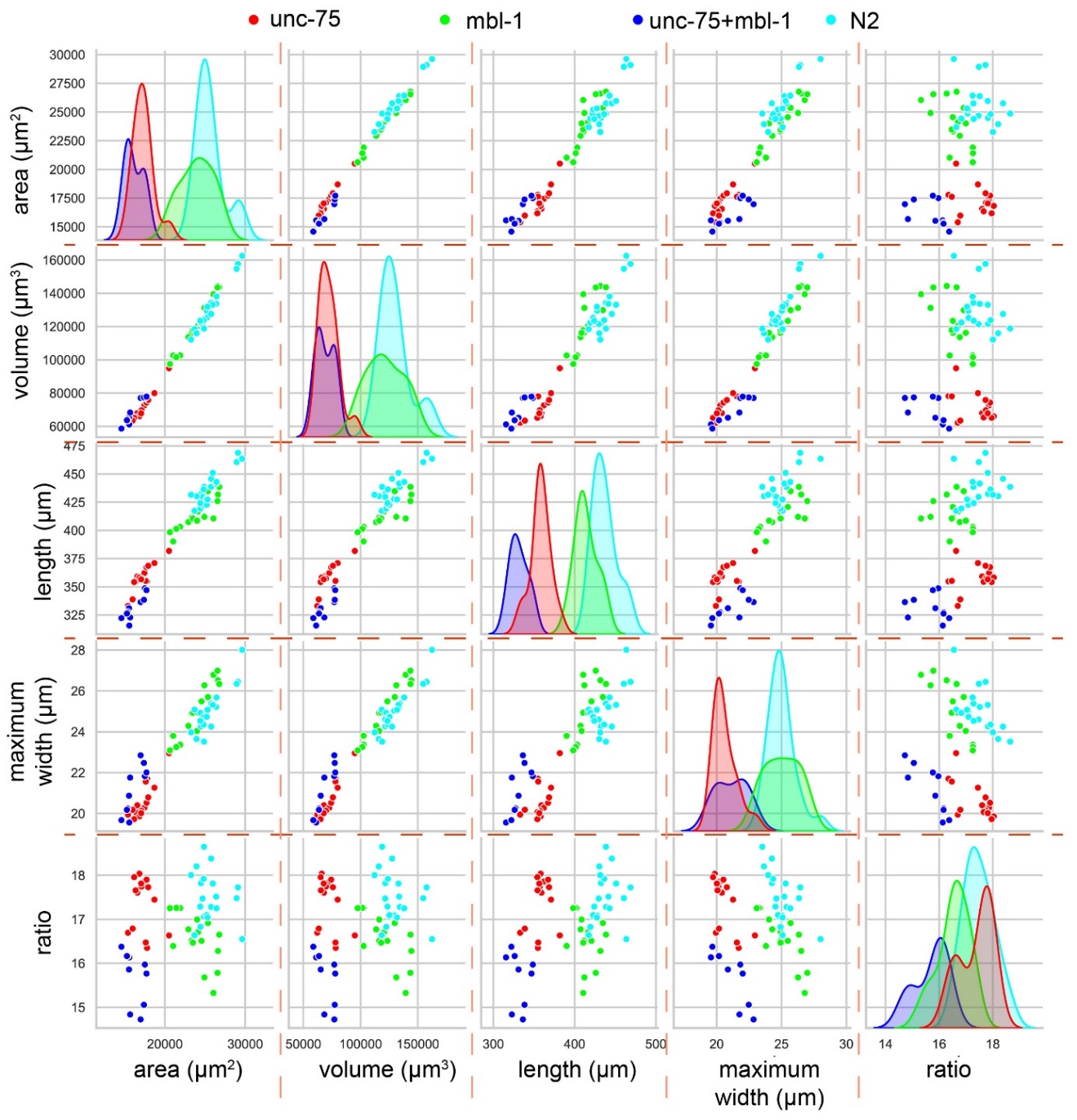


Fig. S27. Correlation between different morphological metrics of young adults from different strains.


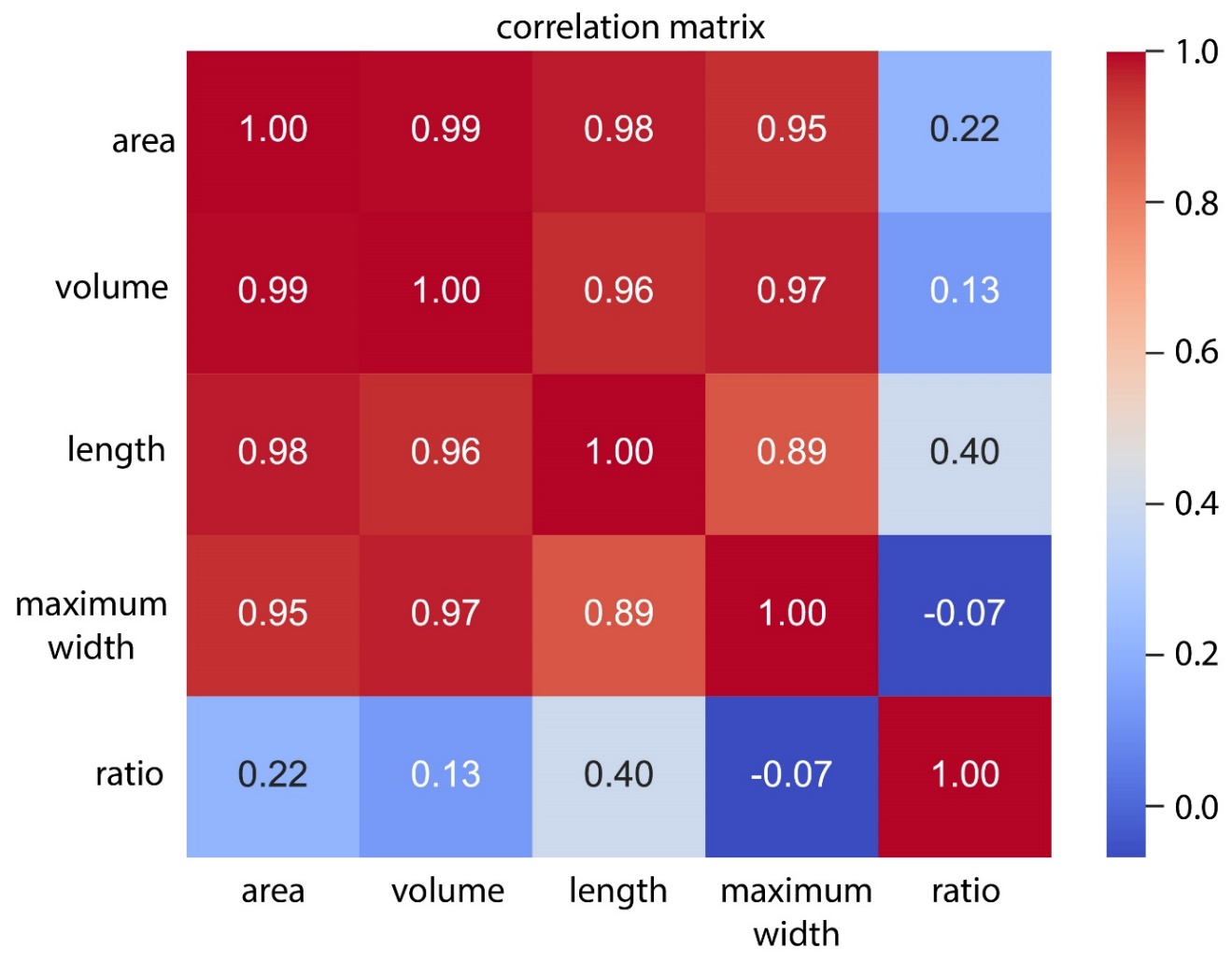


Fig. S28. Correlation matrix between different morphological metrics of young adults from different strains.


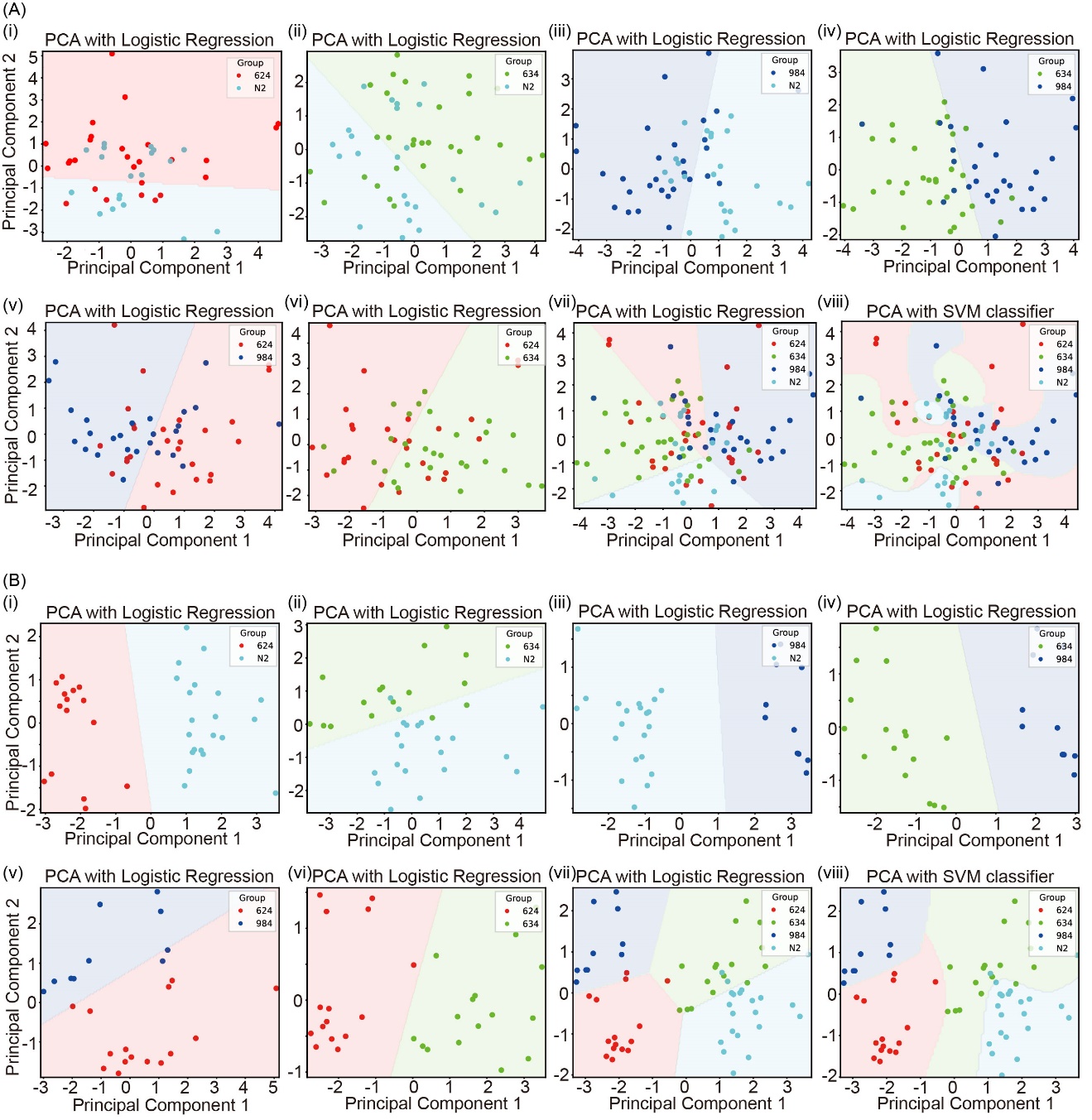


Fig. S29. Classification of embryos and young adults based on the measured morphological metrics of different strain. (A) Classification of embryos: (i)-(vi) classification of embryos from two different strains based on the logistic regression. (vii) classification of embryos from all strains based on the logistic regression. (viii) classification of young adults from all strains based on the SVM classifier. (B) Classification of young adults: (i)-(vi) classification of young adults from two different strains based on the logistic regression. (vii) classification of young adults from all strains based on the logistic regression. (viii) classification of young adults from all strains based on the SVM classifier.


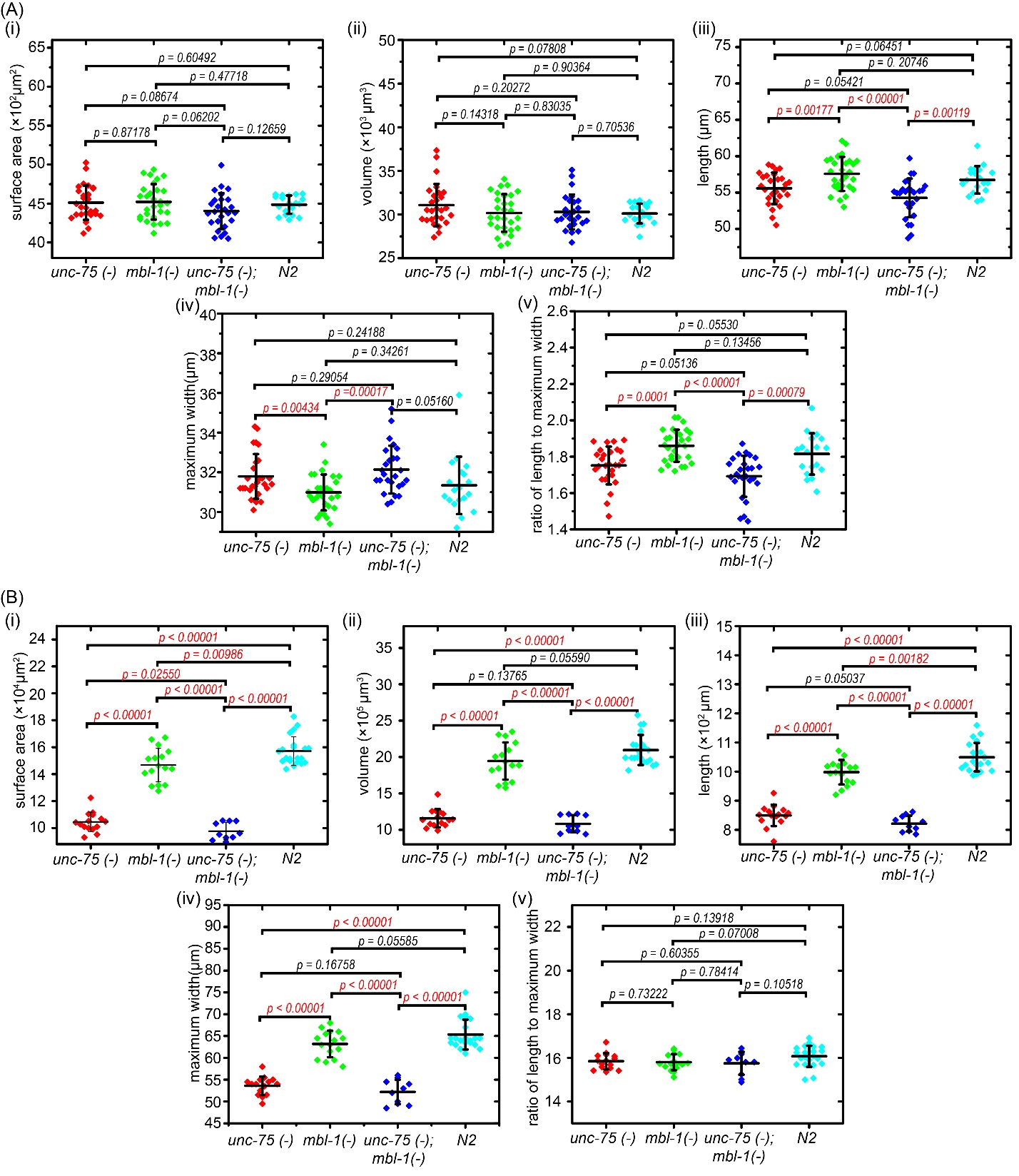


Fig. S30. Morphological phenotyping of the RNA binding protein mutants and N2 relying solely on the contours extracted from a randomly selected 2D image of each sample. (A) Quantification of five key morphological metrics of embryos from different strains: (i) surface area measurement, (ii) volume measurement, (iii) length measurement, (iv) maximum width measurement, (v) ratio of length to maximum width (B) Quantification of five key morphological metrics of young adults from different strains: (i) surface area measurement, (ii) volume measurement, (iii) length measurement, (iv) maximum width measurement, (v) ratio of length to maximum width.

Table S1. Method for the alignment of adults and embryo.


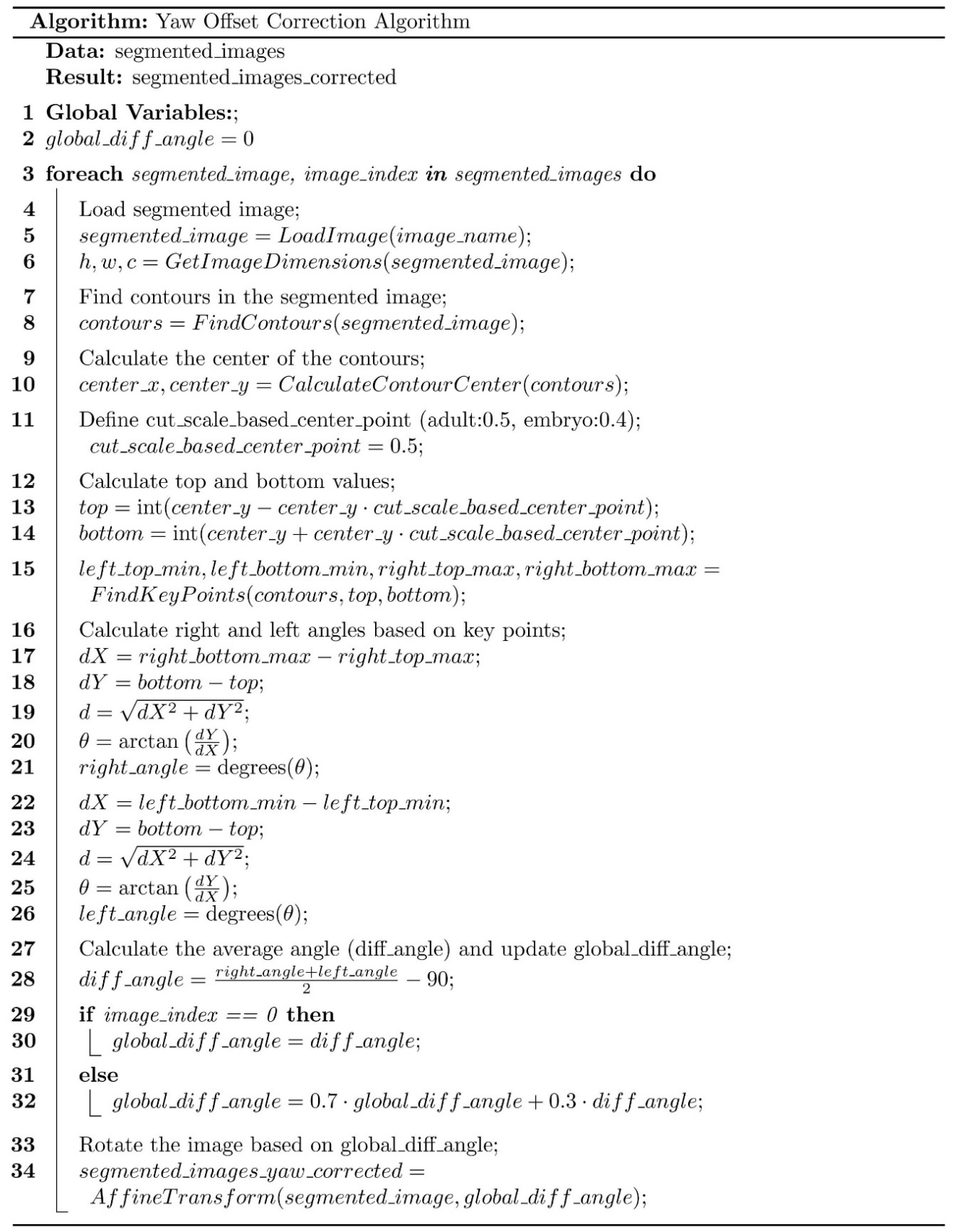


Movie S1. Rotation of single embryo under 20× objective.

Movie S2. Simultaneous rotation of two embryos under 20× objective.

Movie S3. Rotation of adult worms under 4× objective.

Movie S4. Simultaneous rotation of two adult worms under 4× objective.

Movie S5. Rotation of worm at L4 stage under 4× objective.

Movie S6. Rotation of worm at L3 stage under 4× and 10× objectives.

Movie S7. Rotation of worm at L2 stage under 10× objectives.

Movie S8. Stable stoppage of worm rotation via moving substrate leftwards quickly.

Movie S9.

(Movie 9-1): AI based 3D reconstruction of embryos.

(Movie 9-2): 3D models of embryo.

(Movie 9-3): 3D models of different embryos.

Movie S10. AI based 3D reconstruction of adult worms.

**SI References**

1. I. M. Milanovic, K. J. Hammad, Piv study of the near-field region of a turbulent round jet. *Am. Soc. Mech. Eng. Fluids Eng. Div. FEDSM* 1, 1353–1361 (2010).
2. A. Ahmed, A.- Rahman, A. Abdel-Rahman, A Review of Effects of Initial and Boundary Conditions on Turbulent Jets. *Artic. WSEAS Trans. Fluid Mech*. 5, 257–275 (2010).
3. C. Mou, et al., Metric learning based interactive modulation for real-world super-resolution in *European Conference on Computer Vision*, (2022), pp. 723–740.
4. N. Ma, X. Zhang, J. Sun, Funnel activation for visual recognition in Computer Vision--ECCV *2020: 16th European Conference, Glasgow, UK, August 23--28, 2020, Proceedings, Part XI* 16, (2020), pp. 351–368.
5. Y. Yuan, X. Chen, J. Wang, Object-contextual representations for semantic segmentation *in Computer Vision--ECCV 2020: 16th European Conference, Glasgow, UK, August 23--28, 2020, Proceedings*, Part VI 16, (2020), pp. 173–190.
6. A. Kanazawa, S. Tulsiani, A. A. Efros, J. Malik, Learning category-specific mesh reconstruction from image collections in *Proceedings of the European Conference on Computer Vision (ECCV)*, (2018), pp. 371–386.
7. S. Wu, T. Jakab, C. Rupprecht, A. Vedaldi, Dove: Learning deformable 3d objects by watching videos. *Int. J. Comput. Vis.* 131, 2623–2634 (2023).
8. R. B. Rusu, Z. C. Marton, N. Blodow, M. Dolha, M. Beetz, Towards 3D point cloud based object maps for household environments. *Rob. Auton. Syst.* 56, 927–941 (2008).
